# Quantitative *in vivo* Analyses Reveal Calcium-dependent Phosphorylation Sites and Identifies a Novel Component of the *Toxoplasma* Invasion Motor Complex

**DOI:** 10.1371/journal.ppat.1002222

**Published:** 2011-09-29

**Authors:** Thomas Nebl, Judith Helena Prieto, Eugene Kapp, Brian J. Smith, Melanie J. Williams, John R. Yates, Alan F. Cowman, Christopher J. Tonkin

**Affiliations:** 1 The Walter and Eliza Hall Institute of Medical Research, Melbourne, Australia; 2 The Department of Medical Biology, University of Melbourne, Melbourne, Australia; 3 Department of Chemical Physiology, The Scripps Research Institute, La Jolla, California, United States of America; 4 Joint Proteomics Facility, The Ludwig Institute for Cancer Research and the Walter and Eliza Hall Institute, Victoria, Australia; University of Michigan, United States of America

## Abstract

Apicomplexan parasites depend on the invasion of host cells for survival and proliferation. Calcium-dependent signaling pathways appear to be essential for micronemal release and gliding motility, yet the target of activated kinases remains largely unknown. We have characterized calcium-dependent phosphorylation events during *Toxoplasma* host cell invasion. Stimulation of live tachyzoites with Ca^2+^-mobilizing drugs leads to phosphorylation of numerous parasite proteins, as shown by differential 2-DE display of ^32^[P]-labeled protein extracts. Multi-dimensional Protein Identification Technology (MudPIT) identified ∼546 phosphorylation sites on over 300 *Toxoplasma* proteins, including 10 sites on the actomyosin invasion motor. Using a Stable Isotope of Amino Acids in Culture (SILAC)-based quantitative LC-MS/MS analyses we monitored changes in the abundance and phosphorylation of the invasion motor complex and defined Ca^2+^-dependent phosphorylation patterns on three of its components - GAP45, MLC1 and MyoA. Furthermore, calcium-dependent phosphorylation of six residues across GAP45, MLC1 and MyoA is correlated with invasion motor activity. By analyzing proteins that appear to associate more strongly with the invasion motor upon calcium stimulation we have also identified a novel 15-kDa Calmodulin-like protein that likely represents the MyoA Essential Light Chain of the *Toxoplasma* invasion motor. This suggests that invasion motor activity could be regulated not only by phosphorylation but also by the direct binding of calcium ions to this new component.

## Author Summary

Apicomplexan parasites are a group of obligate intracellular pathogens of wide medical and agricultural significance. Included within this phylum is *Plasmodium spp*, the causative agents to malaria and the ubiquitous parasite *Toxoplasma*, which inflicts disease burden on AIDS patients, transplant recipients and the unborn fetus. No matter the host cell that they target, all apicomplexan parasites must activate invasion upon host cell contact. Calcium-mediated signal transduction pathways modulate this process, yet the molecular processes are largely unknown. Using a range of proteomics approaches we reveal proteins in *Toxoplasma* that are phosphorylated upon calcium signaling, and furthermore, identify phosphorylation sites on a range of proteins that may play crucial roles in regulating parasite motility and microneme secretion. By quantitatively monitoring phosphorylation deposition upon calcium signaling we define putative regulatory domains of GAP45 and MLC1 and further show evidence that the invasion motor potentially more strongly associates upon calcium signaling. We also identified that a new Calmodulin-like protein is part of the invasion motor and this suggests that direct Ca^2+^ binding may also modulate motor activity.

## Introduction

The phylum Apicomplexa is a large group of obligate intracellular parasites of wide medical and agricultural significance. Alone, *Toxoplasma gondii* infects between 30- 80% of people worldwide and is one the most common infectious agents of humans. *Toxoplasma* transmission occurs by exposure to the feces of an infected cat, eating undercooked meat or ingestion of contaminated water harboring oocysts [1]. An ocular infection route is also common and is a leading cause of blindness in some countries [2]. Primary exposure or reactivation of tissue cysts in pregnant women can lead to congenital birth defects and spontaneous abortion, while toxoplasmosis is a common secondary infection of AIDS patients and other immuno-compromised individuals and can lead to death if untreated.

Invasion of host cells by apicomplexan parasites is an obligatory step for their survival and proliferation. Despite each species having a range of host and cell types that they target the intracellular processes governing invasion appear to be largely conserved. *Toxoplasma’s* ease of growth in the laboratory and high genetic tractability has provided researchers with an excellent model for investigating the molecular basis of invasion in related species such as *Plasmodium spp -* the causative agent of malaria. For example, a highly conserved actomyosin-based invasion motor that drives parasite motility and invasion was first identified and has been largely characterized in *Toxoplasma*. At its core the invasion motor consists of a novel class XIV Myosin, MyoA [3] and Myosin Light Chain 1, MLC1 [4] which is anchored into the outer side of the inner membrane complex (IMC) by the Glideosome-Associated Proteins GAP40, GAP45, GAP50 and GAPM’s [5], [6], [7], [8]. Recently, the architecture of the invasion motor has been mapped and the central role and absolute requirement of GAP45 has been demonstrated [7]. Furthermore, it was shown that GAP45 spans the supra-alveolar space providing cohesion between the IMC and plasma membrane [7]. The current model of invasion suggests that upon host cell contact GAP40/45/50, GAPM’s, MyoA and MLC1 complex and filamentous actin forms [9]. Transmembrane host cell adhesins linked to actin filaments through the glycolytic enzyme aldolase are then pulled rearwards by the action of MyoA, thus driving the parasite forward into the host cell [10].

The release of apical organelles is also required for successful invasion. Micronemes are first to be secreted and contain high affinity host cell ligands that are necessary for strong host cell attachment and invasion [11]. Rhoptries, large club-shaped organelles, rich in lipids are released after micronemes upon contact of the apical end of the parasite with the host cell. Rhoptry release is correlated with host membrane penetration and the creation of the parasitophorous vacuole [12]. The molecular machinery that drives exocytosis of these apical organelles is unknown.

Activation of the invasion motor and regulated release of apical organelles occurs after a change in extracellular environment and contact with the host cell [13]. Pharmacologically, calcium-dependent signal transduction pathways have been identified in *Toxoplasma* and *P. falciparum* and appear to be essential for micronemal release, motility and invasion [14], [15]. Calcium mobilizing agents potently induce exocytosis of micronemes and gliding motility, whereas membrane permeable calcium chelators such as BAPTA-AM inhibit these processes [11], [15]. In addition, ethanol induces a transient [Ca^2+^]_i_ increase and micronemal release via a putative signaling pathway involving the activation of phospholipase C (PLC) [16]. Further supporting a role of calcium signaling in apicomplexan parasite invasion is the visualization of intracellular calcium ([Ca^2+^]_i_) oscillations that accompany invasion processes in *Toxoplasma* [14], [17] and *Plasmodium* [15]. Together, this data indicates that [Ca^2+^]_i_ signals mediate multiple signaling pathways, including micronemal release and gliding motility and these are essential for regulating the processes involved in host cell invasion.

In mammalian cells, spatiotemporally distinct [Ca^2+^]_i_ signals are translated into enzymatic activity using a variety of serine/threonine-specific protein kinases and phosphatases, which control the function of downstream effectors by promoting local changes in protein conformation, interactions and activity. In Apicomplexa, a conserved family of plant-like Calcium-Dependent Protein Kinases (CDPKs) has been strongly implicated in mediating several critical Ca^2+^-dependent signal transduction pathways during the complex parasite life cycles [18]. Recent chemical genetic and conditional gene knockout approaches have demonstrated that *Toxoplasma* CDPK1 is an essential regulator of Ca^2+^-dependent micronemal exocytosis [19], [20], whereas *P. falciparum* CDPK1 (PfCDPK1) appears to play a role in regulating red blood cell invasion [21]. Furthermore, PfCDPK1 phosphorylates *P. falciparum* Myosin A Tail Interacting Protein (PfMTIP) and Glideosome-Associated Protein 45 (PfGAP45) *in vitro* [21], [22], which suggests a role in regulating invasion motor function during host cell invasion. Together, this data suggests that CDPK’s play crucial roles in regulating invasion processes, yet the *in vivo* substrates of these kinases remain largely unexplored.

To identify substrates of calcium-dependent kinases we have employed proteomics approaches to obtain a global snapshot of the phosphorylation pattern of *Toxoplasma* proteins following stimulation of Ca^2+^ signaling pathways. This identified targets of Ca^2+^-dependent phosphorylation pathways potentially involved in regulating invasion processes. Phosphorylation sites on *Toxoplasma* GAP45, MLC1 and MyoA were identified and given that their is calcium-dependent phosphorylation deposition on only some sites and the presence of a phospho-tyrosine it appears that the phosphorylation status of the invasion motor is likely regulated by multiple kinases. Further, a novel protein was identified as a new component of the *Toxoplasma* invasion motor that associates more tightly upon calcium signaling suggesting that the invasion motor may also be directly regulated by Ca^2+^ binding.

## Results

### *In vivo* stimulation of intracellular Ca^2+^ signaling pathways triggers phosphorylation of multiple proteins

**Figure 1:**
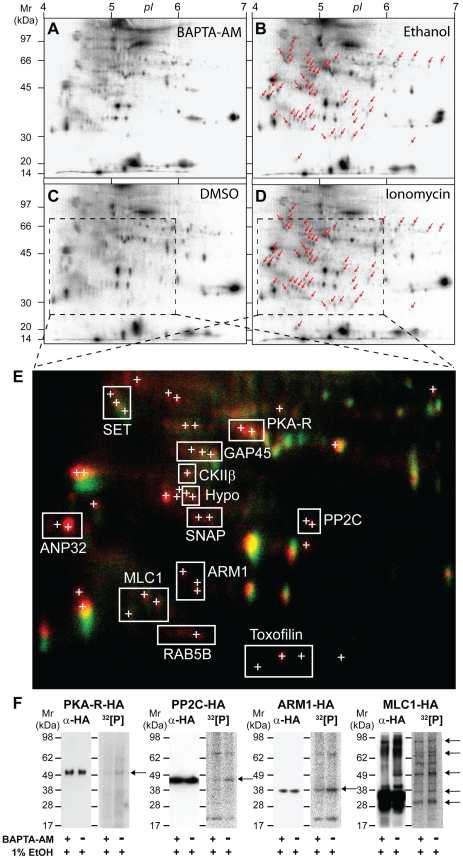
2-DE display of protein phosphorylation events following *in vivo* stimulation of Ca^2+^ pathways in *Toxoplasma gondii* tachyzoites. *Toxoplasma* parasites were incubated in media containing ^32^[P]-sodium orthophosphate to radioactively label intracellular ATP pools and calcium-dependent signal transduction was stimulated *in vivo* by treating free tachyzoites with Ca^2+^-mobilizing drugs, as detailed in Materials & Methods. As negative controls parasites were pre-incubated in the presence of membrane-permeable Ca^2+^-chelator BAPTA-AM (**A**) or treated with DMSO solvent alone (**C**), and for Ca^2+^ pathway stimulation parasites were incubated with 1% (ie. 172 mM) ethanol (B) or 100 µM ionomycin (**D**), respectively. Arrows indicate the positions of 50 ^32^[P]-labeled 2D protein spots that are exclusively phosphorylated following Ca^2+^ signaling pathway stimulation. **E**) A false colour image overlay of corresponding unstimulated (C, green) or stimulated (D, red) autoradiographs showing the relative position of multiple confident LC-MS/MS protein identifications (<1% false protein identification rate). A detailed list of proteins IDs to accompany this figure is provided (Supplementary Table S1). **F**) Radio-immunoprecipitation analyses confirm the phosphorylation of HA-tagged candidate parasite phosphoproteins HA-PKA-R, PP2C-HA, ARM1-HA, MLC1-HA. Free tachyzoites expressing HA-tagged parasite proteins were radioactively labeled with ^32^[P]-sodium orthophosphate and treated with 1% ethanol in the presence (+) or absence of BAPTA-AM (-), as indicated. Anti-HA Western blots (αHA, left panels) and corresponding autoradiographs (^32^[P], right panels) of radio-immunoprecipitates demonstrate a Ca^2+^-dependent increase in the phosphorylation of HA-reactive tachyzoite protein bands (arrows). Abbreviations: PKA-R, cAMP-dependent protein kinase A regulatory subunit; GAP45, Glideosome-Associated Protein 45; CKIIβ, Casein kinase II beta subunit; ANP32, IPP2A-1/ANP32-like protein; SET, IPP2A-2/SET-like protein; SNAP, alpha-soluble NSF attachment protein; PP2C, putative protein phosphatase 2C; ARM1, armadillo repeat-containing protein; MLC1 myosin light chain 1, RAB5B, GTP-binding protein RAB5B; Hypo, hypothetical phosphoprotein.

To identify Ca^2+^-dependent phospho-substrates during *Toxoplasma* invasion we conducted a 2-DE-based screen by labeling parasites with ^32^[P] orthophosphate and stimulating with either ethanol (thought to activate PLC) or the calcium ionophore ionomycin. Both calcium pathway agonists have previously been shown to induce micronemal secretion and motility after short periods of stimulation [16]. As a control parasite samples were either mock treated (DMSO alone) or pretreated with the membrane permeable calcium chelator BAPTA-AM followed by stimulation with ethanol. Extracts were separated on 2-DE gels and over 50 ^32^[P]-labeled protein spots were identified that were absent or barely detectable in BAPTA-AM or mock-treated negative controls (Figure 1A, C) and exclusively present in samples stimulated with either ethanol or ionomycin (Figure 1B, D – marked with red arrows). False colour 2-DE image overlays of corresponding autoradiographs highlight ^32^[P]-labeled protein spots that were specifically phosphorylated following stimulation with Ca^2+^ mobilizing drugs (Figure 1E). Spots that appear yellow likely represent Ca^2+^-insensitive phosphoproteins or GPI-anchored parasite proteins which are unaffected by stimulation, whereas spots that appear red represent potential substrates of calcium-dependent phosphorylation (Figure 1E). Interestingly, the same proteins appeared to be phosphorylated when stimulated by either calcium ionophore or ethanol (Figure 1B, D), strongly suggesting that both agonists stimulate the same signal transduction pathway(s) in *Toxoplasma* tachyzoites.

A total of 50 2-DE spots that were consistently phosphorylated following Ca^2+^ pathway stimulation were subjected to LC-MS/MS-based protein identification (Figures 1E, Supplementary Figure S1 and Table S1). In some instances proteins from separate 2-DE spots shared the same identity, as indicated by bounding boxes (Figure 1E, Table S1). Assuming that phosphorylated 2-DE spots are the product of a single gene the present data identified a total of 63 *Toxoplasma* proteins - some of which represent molecules potentially involved in mediating intracellular signaling cascades, regulating exocytosis of invasion organelles, or controlling parasite motility (Table S1). These included amongst others, a cAMP kinase regulatory subunit (PKA-R, TGME49_042070), a casein kinase subunit II beta subunit (CKIIß, TGME49_072400), a soluble NSF-attachment protein (SNAP, TGME49_018760), an armadillo repeat-containing protein (ARM1, TGME49_061440), a serine/threonine phosphatase 2C (PP2C, TGME49_054770), a Rab GTPase (RAB5B, TGME49_007460), two highly acidic proteins similar to I1PP2A/ANP32 (ANP32; TGME49_071810) or I2PP2A/SET (SET; *TG*ME49_044110), as well as a number of putative uncharacterized proteins (ie. Hypo) (Figure 1E). Importantly, previously identified phosphoproteins were also identified in our analysis including Toxofilin (TGME49_014080), an actin binding protein shown to be phosphorylated by parasitic type 2C phosphatase and CKII activities [23] (Figure 1E, Table S1). Furthermore, we identified ^32^[P]-labeled spots representing GAP45 and MLC1 (Figure 1E, Table S1), indicating that these components of the invasion motor are also likely targets of calcium-dependent phosphorylation *in vivo*.

A limitation in our protein identification methodology is that highly abundant proteins that co-migrate with our calcium-dependent phosphoproteins will be preferentially identified. We therefore sought to confirm the Ca^2+^-dependent phosphorylation of some of the interesting putative phosphoproteins that we identified. To do this we tagged candidates with a C-terminal triple HA tag. Upon radiolabelling parasites and immunoprecipitation with HA antibodies we assessed the phosphorylation status of proteins during calcium stimulation, using ethanol alone or in the presence of BAPTA-AM (Figure 1F). This confirmed that HA-tagged *Toxoplasma* PKA-R, PP2C, ARM1 and MLC1 are *in-vivo* phospho-substrates (Figure 1F). As a control we compared the signal intensity of immunoreactive bands detected on Western blots probed with anti-HA antibody. The ^32^[P]-labeling intensity of the corresponding HA-PKA-R PP2C-HA, ARM1-HA or MLC1-HA protein bands was significantly lower in samples treated with BAPTA-AM prior to stimulation with ethanol (Figure 1F). This is consistent with the increased number and intensity of ^32^[P]-labeled 2-DE spots for native PKA-R, PP2C, ARM1 and MLC1 observed by 2-DE display (Figure 1E) and validates that the phosphorylation of these proteins is dependent on intracellular Ca^2+^ flux.

Focusing in on MLC1 and GAP45 we could see in our 2-DE autoradiographs that both motor components are basally phosphorylated and upon calcium stimulation additional phosphorylated isoforms are detectable (Figure 1E). Multiple ^32^P-labeled protein bands seen in immunoprecipitates from parasite lines expressing HA-tagged MLC1 are consistent with this protein co-purifying with other phosphorylated components of the native invasion motor complex (Figure 1F). Overall, our ^32^[P]/2DE analysis has identified a suite of validated and potential phosphoproteins that respond to calcium signaling and further, identified the *Toxoplasma* invasion motor as a substrate of both calcium-dependent and independent phosphorylation events.

### Identification of phosphorylation sites for *Toxoplasma* calcium-dependent phosphoproteins

**Figure 2:**
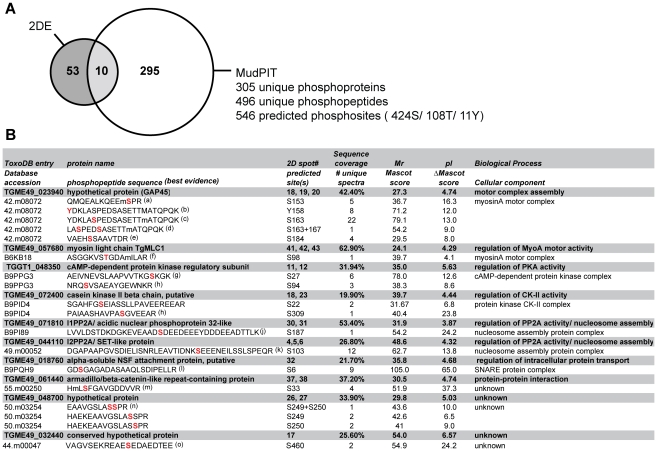
Functional annotation analysis of *Toxoplasma* phosphoproteins identified by MudPIT and comparison of proteomics strategies. A) Venn diagram summarizing the number of confident tachyzoite phosphoprotein identifications (<1% FDR) detected by 2-DE- or MudPIT-based LC-MS/MS analyses. A comparison of 2-DE and MudPIT datasets enabled us to cross-validate 10 major Ca^2+^-dependent tachyzoite phosphoproteins represented by 21 ^32^[P]-labelled 2-DE spots and 86 phosphopeptide spectra (see Supplementary Figure S2, Tables S1-S4 for details). B) Summary of LC-MS/MS evidence for Ca^2+^-dependent tachyzoite phosphoproteins and their associated phosphorylation sites. Listed in the columns (from left to right) are: Top rows (grey background): The ToxoDB entry (ToxoDB release 6.0), protein name, number of 2-DE spot(s) in which each protein was identified (cross-reference to Supplementary Table S1), amino acid sequence coverage, molecular weight (Mr), isoelectric point (pI) and, based on 2-DE gel LC-MS/MS identifications. Bottom row (white background): The database accession (ToxoDB release 4.0 or UniProt entries), predicted phosphorylation site(s), total number of unique spectra (rank 1), highest individual Mascot score (rank 1), delta Mascot score (rank 2), based on LC-MS/MS analyses of MudPIT data. The above information was used in conjunction with independent literature searches to assign keywords associated with Biological Processes or Cellular Components and identify homologs and conserved phosphorylation sites in *P. falciparum*, as indicated in the last column. ^(a-o)^ MS/MS evidence spectra shown in Supplementary Figure S2.

Protein phosphorylation often occurs at low stoichiometry and/or involves proteins with low expression levels. Indeed, some of the proteins identified from ^32^[P]/2DE spots could be more abundant co-migrating proteins that are not responsible for the signal in the autoradiographs. To overcome this issue we employed a Multi-dimensional liquid chromatography Protein Identification Technology (MudPIT)-based strategy for large-scale analysis of phosphorylation sites following *in vivo* Ca^2+^ pathway stimulation of *Toxoplasma* tachyzoites. Proteins were extracted from ethanol-stimulated parasites and digested with trypsin, fractionated via hydrophilic interaction chromatography (HILIC) and partitioned via titanium dioxide (TiO_2_) affinity chromatography. The resulting unbound or phosphopeptide-enriched (bound) fractions were subjected to MudPIT analyses on a LTQ linear ion trap mass spectrometer. Detailed analyses of TiO_2_-bound LC-MS/MS spectra using both Sequest and Mascot-based search algorithms resulted in a total of 305 non-redundant *Toxoplasma* phosphoproteins at 0.9% false discovery rate (FDR) supported by 496 manually approved unique phosphopeptide identifications (Figure 2, Supplementary Tables S2–S4 and Text S1). This data has been integrated into ToxoDB (www.toxodb.org) and will be available in the September 2011 release.

Combining 2-DE analyses of intact ^32^[P]-labeled proteins with global MudPIT phosphoproteomics enabled us to cross-correlate our analysis for the targeted study of parasite proteins modified by phosphorylation following *in vivo* stimulation of Ca^2+^ signaling pathways. This identified 10 major Ca^2+^-dependent tachyzoite phosphoproteins represented by 21 valid phosphoprotein spots and 86 phosphopeptide spectra (Figure 2A). The table in Figure 2B summarises the 2-DE and MudPIT LC-MS/MS data for this subset of the parasite phosphoproteome, which includes 17 putative Ca^2+^-dependent phosphorylation sites. In agreement with differential 2-DE display data (Figure 1E), the existing phosphopeptide MS evidence supports the presence of multiple phosphorylation sites on some phosphoproteins in more than one ^32^[P]-labeled 2-DE spot (Figure 2B and Supplementary Table S1). This includes a cluster of five residues localized on GAP45 (S153, Y158, S163, S167, S184) (Figure S2, A–E) and at least two phosphorylation sites each for PKA-R (S27, S94) (Figure S2, G–H), CKIIß (S22, S309) (Figure S2, I–J), and a hypothetical protein of unknown function TGME49_048700 (S249, S250) (Figure S2, N-O). By contrast, we only detected MS evidence for a single phosphorylation site on MLC1 (S98) (Supplementary Figure S2F), ARM1 (S33) (Supplementary Figure S2M), ANP32 (S187), SET (S103) and SNAP (S6) (Supplementary Figure S2, K–L), most of which were authenticated in multiple ^32^[P]-labeled 2-DE spots (Figures 1E, 2B). We failed to observe any phosphopeptide evidence for PP2C by LC-MS/MS even though our IP analyses of ^32^[P]-labeled parasites expressing transgenic HA-tagged suggested that this protein is a bone fide phosphoprotein (Figures 1F, 2B).

### The *Toxoplasma* invasion motor is heavily phosphorylated

**Figure 3:**
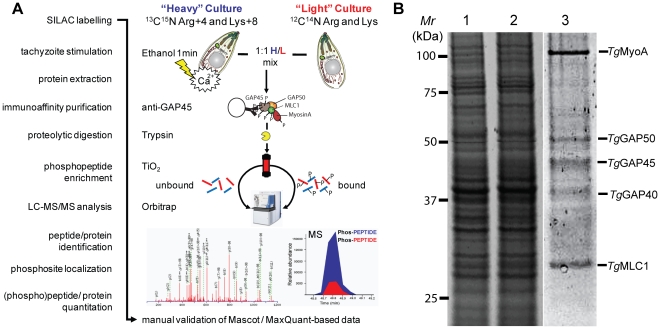
Quantification of calcium-dependent regulation of phosphorylation sites of *Toxoplasma* invasion motor complex components. **A**) Work flow to identify individual phosphorylation sites and quantitatively assess their responsiveness to calcium signals using a SILAC-based proteomics approach. A 1∶1 mixture of Triton X-100 lysates from “Heavy” (H; Arg4/Lys8)-labeled ethanol-stimulated tachyzoites or "Light" (L; Arg0/Lys0)-labeled non-stimulated parasites was generated, and a TiO_2_-enriched phosphopeptide sample of H/L-labeled *Toxoplasma* invasion motor complexes was prepared and analysed by LC-MS/MS on an LTQ-Orbitrap instrument. Mascot and MaxQuant search engines facilitated subsequent manual identification, phosphosite localization and quantification of proteins or peptides as detailed in materials and mathods. **B**) Sypro Ruby-stained SDS-PAGE separation of the relative amounts of light (lane 1) or heavy (lane 2) Triton X-100 whole protein extracts are shown. Intact tachyzoite invasion motor complexes comprising the five major components MyoA, GAP50, GAP45 and MLC1 were precipitated from a 1∶1 H/L mixture by GAP45-specific immuno-affinity chromatography (lane 3).

Complementary 2-DE and MudPIT analyses of whole parasite extracts provided direct evidence that at least 2 of the 5 known myosin motor complex components, namely GAP45 and MLC1, are phosphorylated *in vivo* in a Ca^2+^-dependent manner. To further understand this we employed a Stable Isotope Labeling by Amino Acids in Culture (SILAC)-based proteomics approach and quantitatively assess the *in vivo* phosphorylation of tachyzoite invasion motor complexes following Ca^2+^ pathway stimulation (Figure 3A). First, we demonstrated that over the labeling period we achieved >95% incorporation of ‘heavy’ stably isotopically labeled arginine and lysine into tachyzoite protein, as detailed in Materials & Methods and Supplementary Figure S3. Then we grew SILAC-labeled bulk cultures and prepared protein lysates of un-stimulated (light) or ethanol-stimulated (heavy) tachyzoites. Samples were quantified using Sypro Ruby protein stain (Figure 3B, lanes 1&2), total protein was mixed at a ratio of 1∶1 and used to immunoaffinity-purify *Toxoplasma* myosin motor complexes using anti-GAP45 specific antibodies (Figure 3B, lane 3). As expected anti-GAP45 column eluates were specifically enriched in the five major components of the *Toxoplasma* glideosome complex **–** MyoA, GAP50, GAP45, GAP40 and MLC1 (Figure 3B) [5], [7]. Intact protein complexes were then digested with trypsin and partitioned using TiO_2_-based phosphopeptide enrichment [24]. TiO_2_ eluates and flow through were then run through the high mass accuracy Orbitrap instrument. (Phospho)peptides were then identified and relative abundances calculated using the MaxQuant computational platform [25] (Figure 3A). From this analysis we were able to achieve 70.8%, 36.9%, 52.2%, 3.7% and 73.7% sequence coverage across MyoA, GAP50, GAP45, GAP40 and MLC1, respectively (Supplementary Figure S4).

A total of 61 *Toxoplasma* phosphopeptide sequences were identified in digests of immunoaffinity-purified invasion motor complexes (Supplementary Table S5). They were exclusively singly phosphorylated and recovered in the TiO_2_-bound fraction consistent with a specific enrichment of mono-phosphorylated peptides by TiO_2_ beads [26]. Computational analyses of Orbitrap LC-MS/MS data identified up to 13 potential phosphorylation sites on the invasion motor complex proteins GAP45, MLC1 and MyoA, but none on GAP50 (Table S5). Evaluation of evidence spectra resulted in manual approval of ten unambiguous phosphorylation sites with highly confident Mascot (>25), ΔMascot (>10) and MaxQuant Phospho S/T/Y site probability scores (>90%) (Table 1 and Supplementary Figure S5, A–J). The phosphorylation of neighboring GAP45 residues S184/S185 was the only uncertainty and was narrowed to either site with 50% probability (Table 1 and Supplementary Figure S5E).

**Table 1:** SILAC-based quantitation of changes in the phosphorylation of invasion motor components upon calcium pathway stimulation.

Phospho site	Phosphopeptide (best evidence)	S/T/Y site probability	Mascot score	ΔMascot score	H/L Ratio (normalized)
***GAP45***	***TGME49_023940***				
S153	QMQEALKQEEMSPR ^(a)^	100%	52.5	27.8	1.0 +/− 0.2
Y158	EKYDKLASPEDSASETTMATQPQK ^(b)^	99%	39.0	10.6	1.0 +/− 0.3
S163	YDKLASPEDSASETTMATQPQK ^(c)^	100%	96.9	11.7	1.0 +/− 0.2
S169	YDKLASPEDSASETTMATQPQK ^(d)^	93%	71.8	7.2	1.0 +/− 0.2
S184/185*	VAEHSSAAVTDR ^(e)^	50%	25.5	9.0	3.9 +/− 1.9
T189	VAEHSSAAVTDR ^(f)^	100%	64.8	34.8	3.7 +/− 0.4
***MLC1***	***TGME49_057680***				
S55	VGEYDGACESPSCR ^(g)^	100%	53.3	18.3	1.0 +/− 0.3
T98	VSTGDAMILAR ^(h)^	99%	53.9	10.4	1.6 +/− 0.4
S132	SGDNLDYASFQK ^(i)^	100%	61.1	14.7	1.7 +/− 0.4
***MyoA***	***TGME49_035470***				
S21	RSSDVHAVDHSGNVYK ^(j)^	100%	56.4	12.0	1.3 +/− 0.5

MaxQuant analyses of high mass-accuracy SILAC data reveal that the phosphorylation of at least two sites on *Toxoplasma* GAP45 (S184/185, T189), two sites on MLC1 (T98, S132) and one on MyoA (S21) increased upon calcium pathway stimulation (H/L ratio normalized - underlined). Intact invasion motor complexes were immunoaffinity purified by anti-GAP45 column chromatography, digested in solution using trypsin, and analysed on an Orbitrap LC-MS/MS mass spectrometer as outlined in Figure 3 and detailed in Materials & Methods. Header rows (bold, italics) list the protein name and ToxoDB entry (ToxoDB release 6.0 ID). Listed below, from left to right: The localized phosphorylation site, phosphopeptide amino acid sequence (best evidence), MaxQuant phos(S/T/Y) localization probability, highest individual Mascot score (best evidence, rank 1), ΔMascot score (best evidence, rank 2), and the average H/L ratio and standard deviation of individual phosphopeptide evidence spectra normalized by protein. ^(a-j)^ MS/MS evidence spectra shown in Supplementary Figure S5.

The OrbiTrap SILAC data was further analyzed to quantitate the response of calcium signaling on invasion motor phosphorylation. We calculated the ratio of Heavy:Light phosphopeptides normalized for particular components using the MaxQuant algorithm (Table 1, Supplementary Table S5). This showed that phosphorylation of 4 of the identified residues on GAP45 (S153, Y158, S163, S169) did not change upon calcium stimulation, whereas S184/185 and T189 had H:L ratios of 3.9 and 3.7 respectively (Table 1), strongly suggesting that phosphorylation on these sites is deposited by a calcium-dependent protein kinase (the quantitation of Ca^2+^-dependent GAP45 phosphorylation was manually validated as shown in Supplementary Figure S6, and a detailed discussion of results is provided in Supplementary Text S2). For MLC1 we could determine that while one site (S55) did not respond to calcium signaling, T98 and S132 had mild increases in phosphorylation deposition, again suggesting a role of a calcium-dependent kinase in modulating the post-translationally modified state of this protein (the quantitation of Ca^2+^-dependent MLC1 phosphorylation was manually validated as shown in Supplementary Figure S7 and is discussed in Supplementary Text S3). The only MyoA phosphorylation site that we were able to identify appeared to change negligibly upon calcium stimulation and further work will be needed to identify if S21 phosphorylation truly responds to calcium signaling (Table 1). Given that we identified both serine/threonine and tyrosine phosphorylation and that some residues respond to calcium signaling, while others do not, this suggests that at least three kinases are responsible for the phosphorylation profile of the *Toxoplasma* invasion motor.

### Quantitative proteomics suggests that calcium signaling induces changes in invasion motor assembly

Given that the invasion motor must form a complex to promote parasite motility [7] we wanted to see if we could use our SILAC approach to see what effect calcium signaling has on motor assembly. To do this we analyzed the abundance of non-phosphopeptides from motor complex components and in their different labeling states from anti-GAP45 column eluates. Using our LC-MS/MS analyses on the Orbitrap we identified abundant MyoA, GAP50, GAP45, GAP40 and MLC1 peptides, with a total of 186, 58, 54 or 43 assigned spectra from six independent experiments (including different modifications and/or labeling states) (Table 2 and Supplementary Table S6). Analysis of Heavy:Light ratios of non-phosphopeptides indicated a 2–3 fold increase in the relative abundance of heavy-labeled invasion motor complex components from calcium-stimulated parasites (Table 2 and Supplementary Table S7). By contrast, the average H/L ratio of total *Toxoplasma* protein in these samples was estimated to be 1.1 ± 0.9, as expected given that a 1∶1 mixture of H/L-labeled parasite lysates was used in our pull down experiments (Table 2). This strongly suggests that more invasion motor is available for immunoprecipitation after heavy labeled parasites underwent calcium pathway stimulation. Two possible interpretations of this result are that the invasion motor complex forms more readily upon calcium signaling or the invasion motor could potentially form higher order structures after calcium signaling. This is the first evidence to suggest that changes in the structure or assembly of the invasion motor occurs upon calcium signaling, and until a more in-depth analysis is performed these results should be treated with caution.

**Table 2:** Quantitative assessment of invasion motor complex components.

Protein name	ToxoDB entry	Mr	Sequence Coverage	Unique Peptides	Total Spectra	H/L Ratio	# Total Spectra
MyoA	TGME49_035470	93 kDa	54%	43	186	2.3 +/− 0.9	186
GAP50	TGME49_019320	47 kDa	33%	14	58	2.7 +/− 0.3	58
GAP45	TGME49_023940	43 kDa	50%	12	54	1.8 +/− 0.5	54
MLC1	TGME49_057680	24 kDa	60%	9	43	2.8 +/− 1.0	43
GAP40	TGME49_049850	27 kDa	4%	2	5	3.1 +/− 1.5	5
ELC1	TGME49_069440	15 kDa	87%	11	24	2.4 +/− 0.5	24

Summary of the relative yield of components of immunoaffinity-purified invasion motor complexes. *Toxoplasma* MyoA, GAP50, GAP45, MLC1, GAP40 and a 15 kDa Calmodulin-like hypothetical protein (TGME49_069440) (ELC1-see text) were the five most abundant *Toxoplasma* proteins in digests of unlabeled invasion motor complexes, based on the total number of detected spectra. Quantitative analysis of SILAC-labeled motor complex preparations indicates a specific 2–3 fold enrichment in the yield of heavy-labeled invasion motor complex components following Ca^2+^ stimulation. Intact invasion motor complexes were immunoaffinity purified by anti-GAP45 column chromatography, digested in solution using trypsin, and analysed on an Orbitrap LC-MS/MS mass spectrometer as outlined in Figure 3 and detailed in Materials & Methods. Listed, from left to right: Protein name, ToxoDB entry (ToxoDB release 6.0 ID), theoretical molecular mass (Mr), sequence coverage (%), the number of unique peptides (sequence/modifications/labels), the total number of spectra (total spectra count), and the average H/L ratio for all detected SILAC pairs estimated using the MaxQuant algorithm [51].

### Identification of a Calmodulin-like protein as new component of the *Toxoplasma* MyoA motor complex

By interrogating our SILAC data we were able to show that there are potentially changes in the assembly of the invasion motor upon calcium stimulation. We therefore reasoned that we might also be able to identify other unknown components of this complex by this virtue. The most highly represented additional protein was a hypothetical Calmodulin-like *Toxoplasma* protein with a predicted mass of 15 kDa (TGME49_069440) (Tables 2 and Supplementary Tables S6 and S7), that we have termed Essential Light Chain 1 (ELC1)(see below).

**Figure 4:**
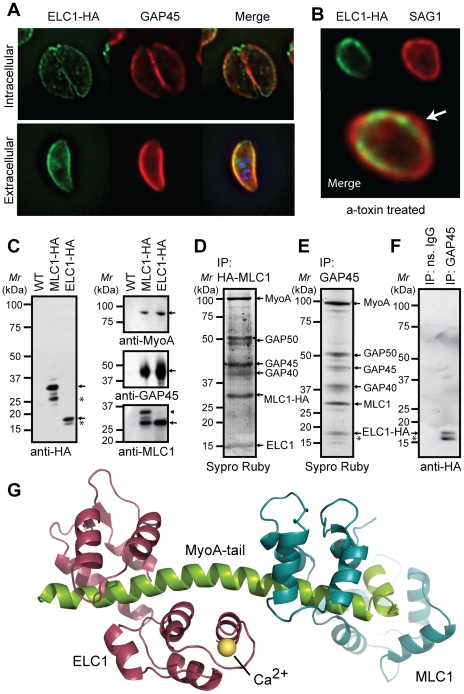
Identification of a potential Essential Light Chain of the *Toxoplasma* myosin motor. (**A**) Immunofluorescence analyses of *Toxoplasma* parasite lines expressing HA-tagged ELC1 confirms the co-localization of this proteins with GAP45 at the parasite periphery, and (**B**) demonstrates a stable association with the inner membrane complex. (**C-F**) Immunoprecipitation analyses validate that ELC1 is an integral component of the intact MyoA invasion motor complex machinery. **C**) Western blot analyses of anti-HA immunoprecipitates prepared from detergent-soluble protein extracts of wild-type (WT) or transgenic parasites expressing HA-tagged MLC1 (MLC1-HA) or ELC1 (ELC1-HA) were probed by Western blot using antibodies against the HA epitope tag, as indicated. Arrows show the relative size of MLC1-HA (∼35 kDa) and ELC1-HA (∼18 kDa) protein bands. We also observed smaller immunoreactive MLC1-HA or ELC1-HA bands in HA pull downs (*). Western blot analyses using specific rabbit polyclonal antibodies against MyoA (top), GAP45 (center) or MLC1 (bottom) confirm the co-purification of other invasion motor complex components in anti-HA immunoprecipitates of parasite lines expressing MLC1-HA (lane 2) or ELC1 (lane 3), but not wild-type controls (lane 1). Arrows show the sizes of endogenous *Toxoplasma* MyoA, GAP45, MLC1, and the size of the immunoreactive band corresponding to MLC1-HA (lane 2, arrowhead). **D**) Immunoprecipitates of parasites expressing MLC1-HA were purified using magnetic microbeads coated with anti-HA antibodies and eluted proteins stained with Sypro Ruby. Major bands corresponding to MyoA, GAP50, GAP45, GAP40, and MLC1 were confirmed by LC-MS/MS. A ∼15 kDa protein band was precipitated in addition to the other known invasion motor complex components and were excised from a preparative 10% SDS-PAGE gel (arrow). This protein band yielded 44% sequence coverage for ELC1 (Supplementary Figure S8). **E, F**) Immunoprecipitates of parasites expressing ELC1-HA were prepared using anti-GAP45 antibodies. Eluted proteins were stained with Sypro Ruby (E) or probed by Western blot using anti-HA antibody (F). A ∼18 kDa protein band corresponding to ELC1-HA was detected in addition to the other known invasion motor complex components (E, arrow). Western blot analyses confirm the co-purification of ELC1-HA with motor complexes prepared from parasite lines expressing HA-tagged ELC1 using specific rabbit polyclonal antibodies against GAP45 (lane 2, arrow), but not in pull-downs prepared using non-specific rabbit IgG (lane 1). An asterisk indicates a putative degradation product, proteolytic fragment or posttranslational modification of ELC1-HA. **G.**) Structural model of *Toxoplasma* MyoA-tail (green) interacting with MLC1 (Cyan) and the newly identified ELC1 (Magenta) based on *P. polycephalum* myosin regulatory complex [34].

To investigate the localization and potential association of this 15 kDa Calmodulin-like protein with the motor complex, we fused this protein with a triple HA epitope tag at the endogenous locus and performed immunofluorescence (IFA) and co-immunoprecipitation/Western blot analyses (CoIP) using anti-HA or anti-GAP45 antibodies (Figure 4). Immunofluorescence microscopy of stably expressing parasites demonstrates this protein resides both at the apical end (likely the conoid) in intracellular parasites and at the parasite periphery as demonstrated by co-localization with GAP45 (Figure 4A). Interestingly, upon egress from host cells extracellular parasites appeared to lose conoid localization and adopt a more typical pattern of an IMC protein (Figure 4A). The significance of this is not yet understood. *Clostridium septicum* alpha-toxin-induced swelling of extracellular parasites revealed this protein is more firmly anchored to the IMC than the plasma membrane (PM), providing further evidence that this protein could be tightly associated with the motor complex (Figure 4B). CoIP’s performed with anti-HA antibody on tagged and wildtype lines (as a control) followed by Western blot analyses formally established the association of this protein with the invasion motor complex (Figure 4C). In addition, pull-downs using antibodies against MLC1-HA (Figure 4D) or GAP45 (Figure 4E, F) precipitated the endogenous or HA-tagged ELC1. The endogenous ELC1 protein was readily detectable as a 15-kDa Sypro-Ruby stained protein band present in anti-HA IP’s in a MLC1-HA expressing line and proven by LC-MS/MS (Figure 4D and Supplementary Figure S8). The co-purification of ELC1-HA by anti-GAP45 antibodies (Figure 4E and Supplementary Tables S6 and 7) was also shown to be highly specific, as demonstrated by IP/Western blot analysis of immunoprecipitates prepared from ELC1-HA expressing parasite lines using anti-GAP45-specific or non-specific rabbit IgG (Figure 4F). Reproducibly, we noticed that this protein could be seen as two bands (Figure 4C, F) but further work will be needed to determine if this is a degredation product or has physiological significance. *Toxoplasma* ELC1 displays limited homology with apicomplexan calmodulins, but has significant structural similarity to the essential light chains of more conventional myosins. We found that the sequences of MLC1 and ELC1 to be highly compatible with the structure of the essential light chain of Scallop myosin II complex (PDB 2BL0, chains C and B, respectively) and therefore we constructed a model of MyoA, MLC1 and ELC1 based on this structure (Figure 4G, protein alignments used are presented in Supplementary ). We therefore suggest that TGME49_069440 is the Essential Light Chain of MyoA (here named ELC1) and given its predicted calcium-binding ability this new component suggests that Ca^2+^ binding could also directly regulate invasion motor activity.

## Discussion

Invasion of apicomplexan parasites is a complex, multistep process critical for the survival of this group of pathogens. In *Toxoplasma* calcium signaling is required for the release of apical organelles and the activation of the invasion motor [18], yet the molecular pathways mediating these processes are unclear. Pharmacological evidence suggests that upon host cell recognition by an unknown ligand, PLC is activated to produce soluble Inositol 1,4,5 triphosphate (IP_3_), which then promotes the release of Ca^2+^ from ER stores. An increase in cytoplasmic Ca^2+^ concentration activates a range of calmodulin and calcium-dependent protein kinases, allowing for phosphorylation of specific targets. This then is thought to change the cellular activity of substrates, thus activating the invasion process [18].

Recent work has identified several kinases that potentially play a role in invasion [18], [19], [21], yet little is known about the substrates that they target. To address this problem we have used global proteomics approaches to identify these substrates and understand the patterns of calcium-dependent phosphorylation upon motility and invasion. We have identified over 50 potential calcium-dependent phosphorylation substrates and detected phosphorylation sites on at least 10 on these proteins. Further, by analyzing the 2-DE pattern of phosphoproteins after stimulation with either ethanol (thought to activate PLC) or the calcium-mobilizing drug Ionomycin we have shown that both these agonists stimulate the phosphorylation of a largely overlapping set of proteins, strongly suggesting that both these agents act to stimulate the same signal transduction cascade. Our analysis therefore provides further evidence that PLC acts in the same pathway as calcium signaling to activate host cell invasion [16], [18].

Using a combination of molecular and biochemical approaches we identified a wide range of *Toxoplasma* phosphoproteins with different predicted cellular functions and validated at least 10 calcium-dependent protein kinase substrates that are phosphorylated. Our work therefore presents a snapshot of the complexity associated with calcium-mediated signal transduction in apicomplexan parasites. It will now be interesting to understand the function of these individual molecules in mediating host cell invasion. For example, does the phosphorylation of the vesicle fusion components αSNAP and ARM1 (homologous to Vac8 from yeast) activate apical organelle release? The calcium-dependent phosphorylation of the kinase regulatory subunits CKIIß and PKA-R and the protein phosphatase PP2C are also interesting observations as they suggest modulation of the activity of these molecules by calcium signal transduction pathways. Dissecting the role of individual calcium-dependent phosphoproteins is clearly the next important step in revealing how this group of pathogens regulates host cell invasion.

**Figure 5:**
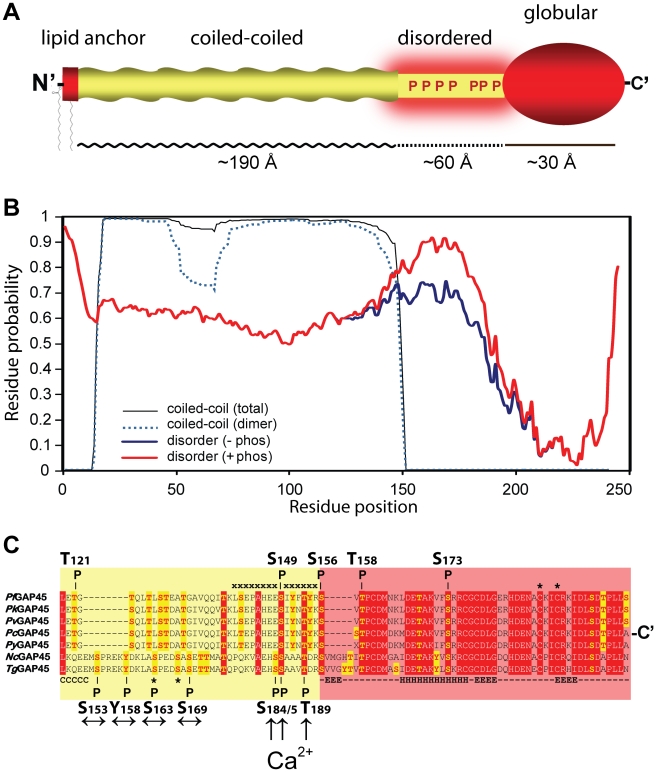
Predicted structure and phosphorylation of *Toxoplasma* GAP45. **A.** Domain model of *Toxoplasma* GAP45 structure. The protein encodes a short lipid anchored N-terminal domain (aa1-15), an extended alpha-helical coiled-coil domain (aa16–151), a region predicted to be intrinsically disordered (aa152–192) and a globular C-terminal domain (aa193–245). The upper limit of the dimension of the coiled-coil, intrinsically disordered and globular domains derived from structural modeling studies are shown. **B.** Prediction of the GAP45 coiled-coil and disordered domains. Plots of the overall probability of GAP45 amino acid residues to form a coiled coil (black line) or form a dimeric coiled-coil (broken blue line) are based on the Multicoils program [54]. Plots of the disorder tendency of the unphosphorylated GAP45 amino acid sequence (blue line, -phos) or GAP45 sequence with glutamate replacement of all S/T/Y residues in region aa152-192 are based on the IUPred algorithm [27]. **C.** ClustalW v.2.1 alignment of GAP45 amino acid sequences from (top to bottom) *P. falciparum* (PFL1090 w), *P. knowlesi* (PKH_143920), *P. vivax* (PVX_123765), *P. chabaudi* (PCAS_143960*), P. yoelii* (PY03448), *Neospora caninum* (NCLIV_048570) and *T. gondii* (TGME49_023940). Highly conserved residues are highlighted in red and potential S/T/Y phosphorylation sites are highlighted in yellow. The positions of PfCDPK1 *in vitro* phosphorylation sites of recombinant PfGAP45 identified by Winter *et al*. (2009) [31](top) and *in vivo* phosphorylation sites localized on *Toxoplasma* GAP45 in this study (bottom) are shown. The sequence of a merozoite-derived PfGAP45 phosphopeptide identified by Green et al [22] and the S163 and S167 phosphorylation sites of TgGAP45 are marked (*)[30], and residues that are part of the coiled-coil (C) alpha helices (H) or beta-sheets (E) are annotated. Arrows indicate Ca^2+^-insensitive (↔) or Ca^2+^-sensitive phosphorylation sites (↑) on TgGAP45 identified in this study.

We have also shown that upon calcium-pathway stimulation the invasion motor is a major *in vivo* target of calcium-dependent phosphorylation. This strongly suggests that calcium-dependent phosphorylation events stimulate invasion motor activity. GAP45 is the major target of phosphorylation with seven identified sites. GAP45 is a multifunctional protein essential for both maintaining cohesion between the IMC and the PM and also for recruiting MyoA-MLC1 to the parasite periphery for productive movement [7]. GAP45 is tethered to the cytoplasmic face of the plasma membrane via it’s N-terminal lipid anchor and to extend across the ∼300 Å supra-alveolar space to the outer face of the inner membrane complex, where it interacts with invasion motor components via it’s C-terminal globular domain [7]. All seven GAP45 phosphorylation sites that we identified are clustered together in a semi-conserved region (aa 152-192) between the predicted coiled-coil region and the highly conserved globular domain (Figure 5A, B). Although it has been demonstrated that the coiled-coil and globular domain are both important for parasite motility [7] nothing is yet known about the role of this highly phosphorylated region. Bioinformatic analysis of the pairwise energy content of the GAP45 amino acid sequence using the IUPred algorithm [27] predicts this region to be intrinsically unstructured (Figure 5B, blue line). Furthermore, when the S/T/Y phosphorylation sites are replaced by glutamic acid (mimicking phosphorylated residues), IUPred predicts an additional ∼25% increase in the disorder tendency of this domain (Figure 5B, red line). Interestingly, clusters of phosphorylation are often located in regions predicted to be intrinsically unstructured [28] and are commonly protein-protein interaction domains [29]. This suggests that GAP45’s hyperphosphorylated region may represent a protein-protein interaction domain regulated by phosphorylation. Indeed, recent studies have implicated phosphorylation of S163 and S167, two calcium-independent phosphorylation sites within this region, in modulating an interaction of GAP45 with GAP50 [30], further supporting the notion that this region is involved in motor regulation through protein interaction.

It is also apparent that only the calcium-dependent phosphorylation sites on *Toxoplasma* GAP45 are conserved amongst apicomplexan species (Figure 5C). Of the three Ca^2+^-dependent residues authenticated in the present study, S184 is replaced by a glutamic acid in sequences of other *Plasmodium* species, *Toxoplasma* GAP45 residue S185 maps to *in vitro* phosphorylation site S149 on PfGAP45 [31], and TgGAP45 residue T189 did not map to any identified PfCDPK1 *in vitro* phosphorylation site (Figure 5C). Homologous Toxoplasma residues corresponding to other PfGAP45 phosphorylation sites identified as *in vitro* substrates of PfCDPK1 (S156, S173) [31] were not detected in our present study, putting some doubt on the relevance of the sites identified *in vitro* (Figure 5C). This data suggests that essential aspects of Ca^2+^-dependent phosphorylation of GAP45 might be highly conserved amongst apicomplexan species (Figure 5C).

**Figure 6:**
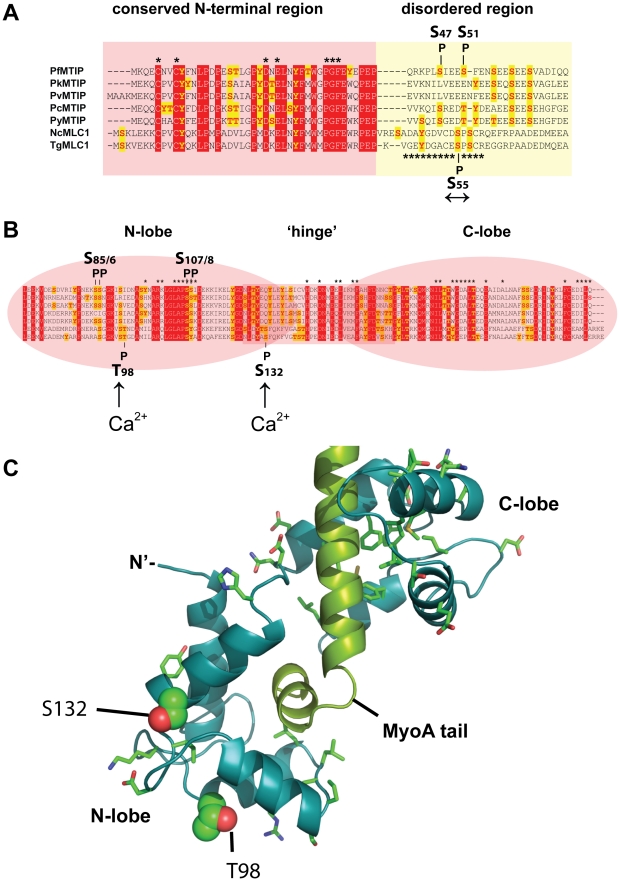
Predicted structure and phosphorylation of *Toxoplasma* MLC1. **A.** ClustalW v.2.1 alignment of the N-terminal sequences of myosin A tail interacting protein (MTIP) from (top to bottom) *Plasmodium falciparum* (PFL2225 w), *P. knowlesi* (PKH_146380), *P. vivax* (PVX_101215), *P. chabaudi* (PCAS_146180*), P. yoelii* (PY00409), and MLC1 protein sequences of *Neospora caninum* (NCLIV_029420) and *T. gondii* (TGME49_057680). The positions of PfMTIP residues S47 and S51 phosphorylated by PfCDPK1 *in vitro* [22] (top), and Ca^2+^-independent TgMLC1 phosphorylation site S55 (↔) localized in this study are shown. Residues that are part of the conserved N-terminal region or intrinsically disordered region of MLC1 are highlighted in red or yellow, respectively. The sequence of a post-translationally modified *Toxoplasma* MLC1 peptide known to inhibit myosin motor activity is marked [32]. **B**) Domain structure and ClustalW alignment of the C-terminal sequences of MTIP/MLC1 proteins. The positions of PfMTIP residues S85/6 and S107/9 phosphorylated by PfCDPK1 *in vitro* [21](top), and Ca^2+^-dependent *Toxoplasma* MLC1 phosphorylation site T98 and S132 (Ca^2+^↑) localized in this study are shown. **C**) Structural model of the *Toxoplasma* MyoA regulatory tail domain bound to MLC1 of GAP45. The positions of the two Ca^2+^-dependent *in vivo* phosphorylation sites T98 and S132 (Ca^2+^↑) are highlighted.

In *Toxoplasma* MLC1 anchors MyoA to GAP45 through a long N-terminal extension (Figure 6A)[7], separate from the degenerate EF hands (Figure 6B). Alignments of MLC1/MTIP’s from other Apicomplexa suggest that this domain can be further broken down into a relatively well-conserved N-terminal portion and a C-terminal region, which, like the hyperphosphorylated segment of GAP45, is also predicted to be intrinsically unstructured (Figure 6A, Supplementary Table S8). The calcium-independent phosphorylation site S55 that we have identified is found within this disordered segment of the N-terminal extension (Figure 6A). Within this region two other phosphorylation sites have been identified *in vitro* in *P. falciparum* [21], [22], and like S55 also appear to be unconserved across the phylum. Another post-translational modification has been identified neighboring phospho-S55 and although the nature of this modification is currently unknown it has been implicated in regulating the activity of the invasion motor [32]. This region of MLC1 therefore appears to be a hotspot of posttranslational modifications, suggesting that this could also be a regulatory domain controlling the activity and/or assembly of the invasion motor. Given the role of the N-terminal segment of MLC1 in anchoring MLC1-MyoA to GAP45 [7] it is possible that S55 and other posttranslational modifications found within this region could regulate this interaction.

The two calcium-dependent phosphorylation sites that we identified on MLC1 map to the C-terminal region, containing the degenerate EF hands responsible for binding the MyoA tail (Figure 6B). The two Ca^2+^-dependent MLC1 phosphorylation sites that were located in this work (T98, S132) do not correspond to residues critical for MyoA binding [33], nor are they conserved amongst Apicomplexa (Figure 6B). To understand a potential role of Ca^2+^-dependent phosphorylation we mapped these sites onto a modeled structure of MLC1-MyoA (Figure 6C). The Calmodulin-like C-terminal region of MLC1 shows significant structural homology to the regulatory domain of scallop (*Physarum polycephalum)* myosin, which is made up of two highly ordered degenerate EF-hand domains (N- and C-lobe), connected by a central helical ‘hinge’ region (Figure 6B, C)[34]. Interestingly, the calcium-dependent phosphorylation sites that we identified map to the N-lobe and on the surface of one particular side of MLC1’s predicted three-dimensional structure (Figure 6C). This poses the interesting possibility that this particular region could be an interaction face regulating the attachment of MLC1 with another invasion motor component.

The only phosphorylation site to be identified on MyoA was at S21. We saw that this site changes mildly in abundance upon calcium signaling. This region is found in the head of the MyoA a region that binds to the actin filament. Although this residue appears conserved across Apicomplexan species (Supplementary Figure S10) we at this stage are unable to speculate on its function.

Upon calcium pathway stimulation we also noticed that another protein more strongly associates with the invasion motor and we show that this Calmodulin-like protein is a *bona fide* component of the *Toxoplasma* motor complex. This Calmodulin-like *Toxoplasma* protein is phylogenetically distinct from both canonical calmodulins and myosin-light chains of apicomplexans [35]. Based on the presence of the intact EF hands of this component and the predictions from protein threading we suggest that this new component could represent the Essential Light Chain of MyoA.

Myosin activity in muscle tissue is regulated by one of two mechanisms, either by the calcium-dependent phosphorylation of the regulatory light chain (RLC) or by the direct binding of calcium ions to an EF hand within ELC. Both RLC and ELC bind to the lever arm of myosin and either mode of activation is thought to rigidify the myosin motor and allow it to function more efficiently [36]. We found that the sequences of MLC1 and ELC1 to be highly compatible with the structure of the essential light chain of scallop myosin II complex (PDB 2BL0, chains C and B, respectively) and therefore we constructed a model of MyoA, MLC1 and ELC1 based on this structure (Figure 4G and Supplementary Figure S9). The model of *Toxoplasma* ELC1 includes a calcium-binding site juxtaposed near the region of closest approach between the ELC1 (residues T16-D17) and MLC1 (residues Y177-G178-E179) – homologues to G178 that is highly conserved amongst RLC’s and other calmodulins. Therefore the model predicts that an interaction between *Toxoplasma* MLC1 and ELC1 is also possible (Figure 4G). This opens the way to investigating the theory that invasion motor activity may be regulated by direct binding of calcium ions as well as calcium-dependent phosphorylation.

What kinases are responsible for invasion motor phosphorylation? We hypothesized that enzymes engaged in these events may be associated with the invasion motor complex. We therefore interrogated our proteomic data set to identify possible interacting kinases, but unfortunately we were unable to detect any significant hits which we attribute to be likely due to the transitory nature of kinases with their substrates. Appraisal of the literature and the motif signature of calcium-dependent phosphorylation sites suggest that members of the CPDK family are the most likely candidates (data not shown). Given that PfCDPK1 has been implicated in motor phosphorylation in *P. falciparum* the most likely candidate in *Toxoplasma* is the phylogenetically related *Toxoplasma* kinase CDPK3 [18]. TgCDPK3, like PfCDPK1, has a predicted motif for N-terminal acylation, which could potentially anchor it to the PM, putting this enzyme in the vicinity to modulate invasion motor phosphorylation in this parasite.

There are several possible kinases that could modulate the calcium-independent phosphorylation status of the motor; Protein Kinase B (PKB) has been shown to phosphorylate PfGAP45 *in vitro*, whereas *Toxoplasma* Protein Kinase G (PKG) is found at the periphery and a specific inhibitor to this enzyme prevents parasite motility and invasion [37]. Another candidate is Casein Kinase II (CKII). The beta regulatory subunit of CKII in *Toxoplasma* also localizes to the periphery of parasite [38] and two calcium-independent phosphorylation sites on GAP45 conform to a CKII-like substrate motif (data not shown).

A tyrosine-based kinase also likely modulates the phosphorylation status of the invasion motor. Tyrosine 158 on GAP45 is phosphorylated in a calcium-independent manner. Given that tyrosine kinases form a unique class of enzyme this suggests a third pathway regulates that invasion motor phosphorylation. Tyrosine kinase and tyrosine phosphorylation have not yet been reported for any apicomplexan parasite, and therefore, it is not possible to speculate on what the identity of this kinase might be. Overall, our data suggests that there are at least three different kinases, and therefore, at least three potential pathways that modulate the phosphorylation status of the invasion motor. Applying specific conditional mutants to our quantitative proteomics approach has the potential to reveal the *in vivo* kinase for these important phosphorylation events.

We have also shown that upon induction of calcium signaling more invasion motor components are found associated with GAP45. This suggests that although the invasion motor can be found pre-formed in resting parasites, calcium signaling induces changes in the association between GAP45 and the other components. Mechanistically such a change could be one of the following: 1. Inducing more invasion motor complexes to form from individual components, 2. The invasion motor complex is rendered more stable after calcium signaling or 3. A higher order structure, such as multimerization is induced. To distinguish from these possibilities and to confirm our finding is physiologically relevant biochemical analyses of isolated invasion motor complexes will need to be performed.

## Materials and Methods

### Cloning of DNA constructs

Predicted open reading frames of phosphoproteins (as described at www.toxodb.org) were amplified and inserted into *Toxoplasma* transfection vectors. *Toxoplasma* MLC1, ARM1 and PP2C were amplified with primers 1&2, 3&4, 5&6 (Table 3) respectively and inserted into *Bgl*II/*Afl*II sites of pCT3H (Tonkin, unpublished), which enabled the tagging of these genes with at the C-terminus with a 3xHA tag. PKA-R was amplified with primers 7&8 and a HA tag introduced by adding this sequence ending this tag onto the 5’ primer (Table 3). PKA-R PCR product was then inserted behind the fkbp-derived destabilization domain (DD) at *Avr*II/*Pst*I of pCTDD (Tonkin, unpublished).

**Table 3:** List of primers used in this study.

Number	Sequence	Features
**1**	AGTCAGATCTATGAGCAAGGTCGAGAAGAAATGCC	*Bgl*II –underlined
**2**	AGTCCCTTAAGCTCCCTTCGCTCGAGCATTGCCTTGC	*Afl*II -underlined
**3**	AGTCAGATCTATGGGGAACCAATGCTGCGCAGGCC	*Bgl*II -underlined
**4**	AGTCCTTAAGCTCCGACAGCCGGACCAAGAGGAGGT	*Afl*II -underlined
**5**	AGTCAGATCTATGAAGTCCTCTGCTGAAATTAGGC	*Bgl*II - underlined
**6**	AGTCCTTAAGATCAGTCTTCTTGAAGAACACTGTC	*Afl*II – underlined
**7**	AGTCCCTAGG *TATCCTTACGATGTTCCAGATTATGCC*CTTAAG ATGCCTCAGCCGAGCGAGGCGTACATGTC	*Avr*II –underlined, HA tag -italicized
**8**	AGTCCTGCAGTTACATATGTTTGTCGAGGTATTTGGTGTC	*Pst*I- underlined

The invasion motor associated Calmodulin-like protein (TGME49_069440) was tagged at the 3’ end of the endogenous gene using ΔKu80 parasite line [39]. A 3’ flank of this gene was amplified with primers 9 and 10 and inserted into pLIC-HA3/HX [40] using the ligation independent cloning strategy. Plasmids were linearized within the gene flank for efficient homologous integration after transfection [39].

### *Toxoplasma* culture and transfection

*Toxoplasma* parasites were grown using standard procedures. Briefly, tachyzoites were grown in confluent human foreskin fibroblasts (HFF) or Vero cells maintained in Dulbecco’s Modified Eagle’s Medium (DMEM; GIBCO, Invitrogen) supplemented with 10% cosmic calf serum (CCS) and an additional 2 mM glutamine (Gibco). If HFF were used DMEM/10%CCS was replaced with DMEM supplemented with 1% fetal calf serum (DMEM/1%FCS) with additional 2 mM glutamine (Gibco) upon infection with parasites.

*Toxoplasma* was transfected using standard procedures [41], [42]. Electroporated tachyzoites were inoculated onto confluent HFF cells and selected on 6 ug/ml of chloramphenicol [41] or Mycophenolic Acid (25 µg/ml)/Xanthine (50 µg/ml) for stable transfectants [43].

### Immunofluorescence analyses and microscopy

IFA was performed using standard procedures. Briefly, Parasites, either intracellular or extracellular, were fixed in 4% paraformaldehyde, permeabilized in 0.1% Triton-X/PBS and blocked in 3% BSA/PBS. Anti-HA mAb (clone 3F10;Roche), anti-GAP45 [5] and anti-SAG1 (a kind gift from L.D Sibley) were decorated onto blocked slides and primary antibodies were detected with antibodies conjugated to AlexaFluor-594 and 488 (Molecular Probes). Extracellular parasites were stuck to slides using 0.1% polyethylenimine. Parasites were treated with dialyzed *C. septicum* culture supernatant containing alpha-Toxin for ∼3 hours. Parasites were then imaged on a Zeiss Inverted Axioscope equipped with AxioCam MRM and Axiovision with Deconvolution software.

### Radioactive or stable-isotope labeling and Ca^2+^ pathway stimulation of *Toxoplasma*

For radioactive or heavy isotope labeling and Ca^2+^ pathway stimulation studies, specialized cell culture conditions were applied. For radioactive labeling, Vero cells were infected with *T. gondii* tachyzoites and metabolically labeled in 25 ml of custom sodium phosphate-free DMEM (GIBCO, Invitrogen) supplemented with 10% fetal calf serum, 2 mM glutamine and 20 µCi/ml of ^32^[P]-labeled monosodium phosphate, overnight.

For stable isotope labeling studies, HFF were infected with *Toxoplasma* and grown in custom DMEM medium devoid of lysine, arginine and leucine. This media was then supplemented with 1% fetal calf serum (dialyzed against sterile PBS), 2 mM glutamine, 0.802 mM L-leucine, and either 0.398 mM L-Arginine + 0.798 mM L-Lysine for the „light“ condition (R0/K0 media), or 0.398 mM (^15^N_4_) L-Arginine + 0.798 mM (^13^C_6_, ^15^N_2_) L-Lysine (98% isotopic purity; Sigma-Isotec) for the “heavy” condition (R4/K8 media). Heavy label incorporation was applied to parasites for ∼6 days to get maximal stable isotope incorporation before bulking up and subsequent parasite harvest. Analysis demonstrated >95% incorporation of R4 or K8 labels after this time (Supplementary Figure S3).

The preparation of free, viable tachyzoites was done as follows; Upon the >80% disruption of HFF cells, parasites were scraped, needle passed and pushed through a 5 µM pore-size membrane filters (Millipore) and then repeatedly washed in ice cold Invasion Medium (DMEM/HEPES/1%FCS) and counted using a hemocytometer. Aliquots of radioactive or heavy-isotope-labeled tachyzoites were resuspended in invasion medium and incubated at 37°C for 10 min prior to Ca^2+^ pathway stimulation. *In vivo* Ca^2+^ pathway stimulation was achieved by adding 1% (171 mM) ethanol or 1 µM ionomycin. As a control, samples of parasites were either mock treated (equal volume DMSO) or were pretreated for 10 minutes with the membrane-permeable calcium chelator BAPTA-AM, followed by stimulation with 1% ethanol. Samples were stimulated for 60 seconds, immediately mixed with 0.5 ml ice cold 2 × lysis buffer (40 mM HEPES pH 7.4, 300 mM NaCl, 2% Triton X-100, 1% NP-40) containing protease and phosphatase inhibitors (HALT protease and phosphatase inhibitor solution, PIERCE) and snap frozen in liquid nitrogen.

### Preparation of parasite protein extracts

Parasites were disrupted by freeze thawing and ultrasonication in ice cold lysis buffer (20 mM HEPES pH 7.4, 150 mM NaCl, 1% Triton X-100, 0.5% NP-40) including protease and phosphatase inhibitors (Pierce), and proteins extracted for 30 min on ice with vortexing. Parasite extracts were clarified by ultracentrifugation at 75,000 g for 30 min and the detergent-soluble protein fraction was precipitated using 2-D Clean-Up kit (GE-healthcare).

### 2-D gel electrophoresis

For 2-DE gel electrophoresis, precipitated *Toxoplasma* protein preparations (∼50 µg protein for analytical gels/∼500 µg protein for preparative gels) were redissolved in 300 µl rehydration/sample buffer (7 M Urea, 2 M Thiourea, 2% ASB-14, 1% DTT, 1% ampholytes), loaded onto 13 cm pI 4–7 IPG strips by passive rehydration and focused at a current limit of 50 µA/IPG strip using a fast voltage gradient (8000 V max, 24,000 Vh) at 15°C. The second dimension was carried out on 10% polyacrylamide gels (18 cm×16 cm×1.5 mm) using a Hoefer SE 600 system (GE Healthcare) at 75 V constant voltage and 10°C overnight. Analytical 2-DE gels were electrophoretically transferred to Immobilon-PSQ PVDF membranes (Millipore). Protein spots on PVDF membranes were visualized using Deep Purple protein stain (GE Healthcare), and protein spots in preparative 2-DE gels were stained with Sypro Ruby Protein stain (Molecular Probes), according to manufacturer’s protocols. Imaging of ^32^[P]-labeled protein spots was achieved by direct autoradiography (7 day exposure) of dry PVDF membrane blots using FUJIFILM BAS-TR2040 tritium imaging plates. Fluorescent or autoradiographic 2-DE images were digitized on a FLA-3000 laser-scanning detection system (Fuji) and manually matched and annotated using Image Master Platinum v7.0 2D image analysis software (GE Healthcare). Preparative 2-DE gels were counter-stained using Colloidal Coomassie Brilliant Blue (Sigma) and regions matching ^32^[P]-labeled protein spots manually excised and subjected to LC-MS/MS analysis.

### Gel excision, in-gel digestion and Nano-LC-MS/MS

For 1-DE or 2-DE samples, protein spots or bands were manually excised from preparative SDS-PAGE gels and subjected to automated in-gel reduction, alkylation, and tryptic digestion using a MassPREP Station (Micromass, UK). All gel samples were reduced with 10 mM DTT (SIGMA) for 30 min, alkylated for 30 min with 50 mM iodoacetic acid (SIGMA) and digested with 375 ng trypsin (Promega) for 16 hrs at 37°C. The extracted peptide solutions were then acidified (0.1% formic acid) and concentrated to approximately 10 ul by centrifugal lyophilisation using a SpeedVac AES 1010 (Savant). Briefly, extracted peptides were injected and fractionated by nanoflow reversed-phase liquid chromatography on a nano LC system (1200 series, Agilent) using a nanoAcquity C18 150 mm×0.15 mm I.D. column (Waters) developed with a linear 60-min gradient with a flow rate of 0.5 µl/min at 45°C from 100% solvent A (0.1% Formic acid in Milli-Q water) to 100% solvent B (0.1% Formic acid, 60% acetonitrile, 40% Milli-Q water). The nano HPLC was coupled on-line to an LTQ-Orbitrap mass spectrometer equipped with a nanoelectrospray ion source (Thermo Fisher Scientific) for automated MS/MS. Up to five most intense ions per cycle were fragmented and analysed in the linear trap, with target ions already selected for MS/MS being dynamically excluded for 3 min.

### Large-scale phosphopeptide purification and MudPIT analysis

For proteome-wide phosphopeptide analyses using MudPIT, precipitated *Toxoplasma* protein preparations (2.5 mg of protein of ionomycin-stimulated tachyzoites) were dissolved in a total volume of 1.25 ml of 6 M Urea, 100 mM Tris-HCl (pH 8.0) and sequentially reduced with 5 mM DTT and alkylated with 10 mM iodoacetamide. Samples were diluted 1∶5 (v/v) in digestion buffer (40 mM ammonium bicarbonate, pH 8, 10% acetonitrile, 1 mM Ca_2_Cl) and digested with LysC (Roche) and trypsin (Sigma). Approximately 1 mg tryptic digest was desalted using Sep-Pak C18 6 cc/1 g cartridges (Waters) and fractionated by hydrophilic interaction chromatography on a TSKgel Amide 80 column (TOSOH Biosciences) using an optimized phosphopeptide gradient, as per published protocol [44]. Ten phosphopeptide fractions were collected and lyophilized. Fractions 1-4 were pooled and the resulting 7 samples were resuspended in 100 µl TiO_2_ loading/wash buffer (1 M glycolic acid, 80% ACN, 5% TFA) and purified on self-packed TiO_2_ micro-columns using a highly efficient method for the selective enrichment of phosphorylated peptides, as detailed elsewhere [45]. The flow-through and all washings were combined and constituted the phosphopeptide-depleted (TiO_2_-unbound) fraction. Individual TiO_2_-bound phosphopeptide fractions were analyzed by multi-dimensional LC-MS/MS on an LTQ linear ion trap mass spectrometer (Thermo Scientific), according to published protocols [46].

### Mass spectra database searching

MudPIT LC-MS/MS spectra were analyzed with SEQUEST 2.7 [47] using a non-redundant protein decoy database (Ludwig NR_Q309_con reverse; 9870917 entries for all species). The SEQUEST outputs were analyzed by DTASelect 2.0.37 [48]. DTASelect 2.0 uses a quadratic discriminant analysis to dynamically set XCorr and DeltaCN thresholds for the entire data set to achieve a user-specified false positive rate (1% in this analysis). The false positive rates were estimated by the program from the number and quality of spectral matches to the decoy database [49]. After filtering the results from SEQUEST using DTASelect, MS/MS spectra were analysed using the DeBunker algorithm for automatic validation of phosphopeptide identifications from tandem mass spectra [50]. This software package uses a support vector machine binary classifier to assess the correctness of phosphopeptide/spectrum matches.

For protein identification of gel samples and the identification of additional MudPIT phosphopeptide sequences (ie. not included in the LudwigNR database) LC-MS/MS data were searched against a redundant protein decoy database comprising sequences from the latest version of Swiss-Prot (Human, Bovine, *Plasmodium*, *Toxoplasma* species), Trembl (*Toxoplasma* entries), PlasmoDB/ToxoDB (*Toxoplasma* entries), as well as their reverse sequences (Toxoplasma_decoy; 117496 entries). Mass spectra peak lists were extracted using extract-msn as part of Bioworks 3.3.1 (Thermo Fisher Scientific) linked into Mascot Daemon (Matrix Science, UK). The parameters used to generate the peak lists for the LTQ Orbitrap were as follows: minimum mass 400; maximum mass 5000; grouping tolerance 0.01 Da; intermediate scans 1; minimum group count 1; 10 peaks minimum and total ion current of 100. Peak lists for each nano-LC-MS/MS run were used to search MASCOT v2.2.04 search algorithm (Matrix Science, UK) provided by the Australian Proteomics Computational Facility (www.apcf.edu.au). The search parameters consisted of carboxymethylation of cysteine as a fixed modification (+58 Da, for gel samples only), with variable modifications set for NH_2_-terminal acetylation (+42 Da) and oxidation of methionine (+16 Da), phosphorylation of serine, threonine or tyrosine (+80 Da). A precursor mass tolerance of ±3 Da (LTQ spectra) or 20 ppm (Orbitrap spectra), #13C defined as 1, fragment ion mass tolerance of ±0.8 Da, and an allowance for up to three missed cleavages for tryptic searches was used.

### Generation of anti-GAP45 immunoaffinity resin

To generate immunoaffinity resin anti-GAP45 IgG was purified using a 1 ml HiTrap Protein-A HP column (GE Healthcare) using an ÄKTA prime FPLC chromatography system (Pharmacia). Purified IgG was desalted using a PD-10 buffer exchange column and chemically cross-linked to CN-Br-activated Sepharose 4B resin (GE Healthcare) in 0.1 M NaHCO_3_, 0.5 M NaCl pH 8.5 for 2 h at room temperature. Un-reacted sites were blocked with 1 M triethanolamine pH 8.0 for 2 h at room temperature and the resin stringently washed with 50 mM glycine-HCl, 0.5 M NaCl pH 3.5 and 50 mM Tris-HCl, 0.5 M NaCl pH 8.0 and resuspended in PBS.

### Immunoaffinity purification of *Toxoplasma* invasion motor complexes

Large-scale immunoaffinity purification of intact MyoA motor complexes from *Toxoplasma* parasites was carried out using anti-GAP45 rabbit serum, as previously described [5]. For our SILAC-based quantitative LC-MS/MS analyses, *Toxoplasma* tachyzoites were grown in confluent HFF’s in “light” R0/K0 media or “heavy” R4/K8 media for 48 h. We found that 4×T150 cm^2^ flasks each of light- or heavy-labeled *Toxoplasma* cultures yielded sufficient material to obtain >60% sequence coverage for most of the complex components. Free, viable tachyzoites were prepared and the heavy isotope-labeled parasites were stimulated with 1% ethanol to measure the effect of Ca^2+^-pathways stimulation, as detailed above. Freshly prepared parasite lysates of un-stimulated (light) or 1% ethanol-stimulated (heavy) tachyzoites were prepared as detailed above, mixed at a protein ratio of 1∶1 in 2 ml lysis buffer with protease and phosphatase inhibitors and incubated with 0.25 ml of the anti-GAP45 resin for 1 h at 4°C. Unbound protein was removed and the resin washed 5 times with 1 ml lysis buffer and 2 times with 0.5 ml water by centrifugation at 2000 g for 5 min in 1 ml microcentrifuge spin columns (Pierce). Invasion motor complexes were eluted with 200 µl 0.1% TFA in water and the eluted material dried in a SpeedVac.

### In-solution digestion of *Toxoplasma* invasion motor complexes and phosphopeptide enrichment

Dried samples of anti-GAP45 column eluates were dissolved in a total volume of 250 µl of 6 M Urea, 50 mM ammonium bicarbonate (pH 8.0) and sequentially reduced with 10 mM DTT and alkylated with 55 mM iodoacetamide. Samples were diluted 1∶5 (v/v) in digestion buffer (40 mM ammonium bicarbonate, pH 8, 10% acetonitrile, 1 mM Ca_2_Cl) and digested with proteomics-grade trypsin (Sigma) at 37°C overnight. The resulting peptide solutions were vacuum-dried, peptides resolved in 0.2%TFA in water, desalted on MacroSpin C18 columns (The Nest Group Inc.), eluted with 0.2%TFA/60% ACN in water and again dried in a SpeedVac. Desalted peptide samples were resuspended in 100 µl TiO_2_ loading buffer (1 M glycolic acid in 80% ACN, 5% TFA) and affinity purified using self-packed TiO_2_ microcolumns containing ∼10 µg of 5 µm Titansphere TiO_2_ beads (GL Sciences), using a published method [24]. The flow-through and all washings were combined and constituted the phosphopeptide-depleted (TiO_2_-unbound) fraction. The TiO_2_ eluate comprising the phosphopeptide enriched fraction and the phosphopeptide-depleted fraction were vacuum-dried. Both fractions were redissolved in 0.2%TFA in water, desalted on MacroSpin C18 columns (The Nest Group Inc.) and again dried in a SpeedVac. For LC-MS/MS analyses, dried (phospho)peptide fractions were redissolved in 1 µl 100% formic acid and diluted to 20 µl in MilliQ water. Digested (phospho)peptides were then subjected to nano-LC-MS/MS on an LTQ-Orbitrap instrument (Thermo Scientific), as described above.

### SILAC-based phosphopeptide identification and quantification

LTQ-Orbitrap LC-MS/MS data were searched against the Toxoplasma_decoy database using the MASCOT search engine, as detailed above. Search parameters were identical, except that additional variable modifications were set for heavy-isotope labeling of arginine (+4 Da) or lysine (+8 Da) and the precursor mass tolerance was 20 ppm for the SILAC/Orbitrap datasets. Mascot search results were loaded into MaxQuant (v1.1.08) [51] for peptide and protein quantification as well as Scaffold (v3.0) (www.proteomesoftware.com) for manual validation of peptide spectral matches. Manual validation of all peptide spectral matches was done irrespective of peptide scores or expectation values (E-values) to ensure that all major fragment ions were annotated in accordance with known rules of peptide fragmentation. The Xcalibur program (Thermo Scientific) was used for generating XIC's of selected peptides in order to validate and verify the MaxQuant results.

### Co-immunoprecipitation of invasion motor complexes and Western blot analyses

For anti-HA CoIP’s, freshly released tachyzoites expressing wild-type (negative control) or HA-tagged parasite proteins were harvested as above, washed in PBS, and lysed in ice cold lysis buffer (20 mM HEPES pH 7.4, 150 mM NaCl, 1% Triton X-100, 0.5% NP-40) containing protease and phosphatase inhibitors (Pierce). Protein was extracted for 30 min on ice with vortexing and centrifuged at 50,000 g for 30 min at 4°C. Supernatants were mixed with monoclonal anti-HA antibody coupled to paramagnetic MicroBeads (Miltenyi Biotec) and incubated for 1 h at 4°C. Supernatants were then subjected to CoIP, according to a manufacturer’s protocol for the specific isolation of HA-tagged proteins utilizing a MultiMACS separator (Miltenyi Biotech). For anti-GAP45 CoIP’s, 1 ml protein extracted from parasites expressing ELC1-HA were mixed with 100 µl of ProteinG-coupled paramagnetic microbeads (Miltenyi Biotech), mixed with 5 µl of anti-GAP45-specific rabbit IgG or non-specific rabbit IgG (negative control), and processed as above. For Western blot analyses eluted protein samples were separated by SDS-PAGE on precast 10% or 4-12% NuPAGE Bis-Tris gels, transferred onto Nylon membranes using an iBlot blotting system (Invitrogen), and then probed with specific primary or HRP-conjugated secondary antibody as indicated.

### Protein fold recognition and comparative modeling

Protein fold recognition was conducted using the WURST protein-threading web server [52]. Homology models were constructed using the sequence alignments predicted from WURST with the MODELLER (9v7) comparative modeling software [53].

### List of accession numbers of genes mentioned in the text

PKA-R, TGME49_042070; CKIIß, TGME49_072400; SNAP, TGME49_018760; ARM1, TGME49_061440; PP2C, TGME49_054770; Rab5B, TGME49_007460; ANP32, TGME49_071810; SET, TGME49_044110; Toxofilin, TGME49_014080; Hypothetical protein, TGME49_048700; Conserved hypothetical protein, TGME49_032440; CaM-like (ELC1), TGME49_069440; MLC1, TGME49_057680; GAP45, TGME49_023940; MyoA, TGME49_035470; GAP50, TGME49_019320; GAP40, TGME49_049850.

## Supporting Information

Figure S1 **Matching of ^32^[P]-labeled 2-DE spots for the LC-MS/MS based identification of Ca^2+^-dependent** ***Toxoplasma*** **proteins listed in Table S1. A**) For 2-DE-based identification of phosphorylated proteins upon Ca^2+^ pathway stimulation ^32^[P]-orthophosphate labeled parasites were treated with (red) or without (green) ionomycin. Phosphoproteins were detected via autoradiography and fake coloured for comparison, as detailed in Materials and Methods. **B**) For the MS-based identification of *Toxoplasma* phosphoproteins, 500 µg aliquots of total parasite protein extract were separated on preparative 2D gels and imaged using Sypro Ruby protein stain (Molecular Probes). Fifty parasite phosphoprotein spots that were consistently labeled following Ca^2+^ pathway stimulation were matched, corresponding regions manually excised from preparative 2D gel electrophoresis and subjected to nanoLC-MS/MS analysis, as detailed in Materials and Methods. Please, refer to Supplementary Table S1 for corresponding protein identification results.
(TIF)

Figure S2 **MudPIT (LTQ) MS/MS evidence spectra for phosphopeptides listed in** **Figure 2B** **.** Phosphorylation site localization for the phosphopeptides listed in Table 1. A. GAP45 p153, B. GAP45, pY158 C. GAP45 pS163, D. GAP45 pS163 & pS167, E. GAP45 pS184, F. MLC1 S98, G. PKA-R pS27, H. PKA-R pS94, I. CKIIβ pS309, J. IPP2A/ANP-32 pS187, K. I2PP2A/SET-like pS103, L. SNAP pS6, M. ARM1 pS33, N. Hyp TGME49_048700 pS249 & pS250, O. Hyp TGME49_032440 pS460. The peptide sequences (top left), observed mass (AMU), parent error (ppm), and central regions of the MS/MS spectra (relative intensity vs. m/z) of the [M+2H]^2+^ or [M+3H]^3+^ precursor are shown: Matching b-ions (red) and y-ions (blue) with consecutive neutral losses of phosphoric acid allow a clear-cut annotation in most cases. Prominent parent ion peaks showing neutral loss of phosphoric acid are not shown.
(TIF)

Figure S3 **Quantitative analysis of [^15^N_4_] L-Arginine (R4) or [^13^C_6_,^ 15^N_2_] L-Lysine (K8) incorporation in** ***Toxoplasma.*** For the analysis of [^15^N_4_] L-Arginine incorporation, *Toxoplasma* parasites were grown in ‘heavy’ [^15^N_4_] L-Arginine (R4) or [^13^C_6_,^ 15^N_2_] L-Lysine (K8) SILAC labeling media for 1 or 3 passages, as detailed in Materials & Methods. Heavy label incorporation was applied for up to 6 days in HFF cells before parasite harvest, as indicated. Parasite protein was extracted, digested in solution and analyzed using nano-LC-MS/MS on an LTQ-OrbiTrap instrument. MS data were searched against a *Toxoplasma*_decoy database using the MASCOT search engine, and the Xcalibur program (Thermo Scientific) was used to plot the relative abundance of ‘light (R0) versus ‘heavy’ (R4) arginine-labeled peptides (**A**), or ‘light’ (K0) versus heavy (K8) lysine-labled peptides (**B**). A statistical summary of SILAC label incorporation can be found in panels **C** and **D**.
(TIF)

Figure S4 **Sequence coverage of invasion motor components.** Sequence coverage for MyoA, GAP50, GAP45, GAP40, MLC1 peptides determined by LC-MS/MS analysis of tryptic digest of intact *Toxoplasma* invasion motor complex components (anti-GAP45 column eluates), as shown in Figure 3B. Database accession numbers (ToxoDB v5.0 IDs) and protein name are shown. Identified peptide sequences are highlighted in yellow and modified or labeled residues are shown in green.
(TIF)

Figure S5 **Orbitrap MS/MS evidence spectra for phosphopeptides listed in** **Table 1** **.** Phosphorylation site localization for the GAP45 phosphopeptides QMQEALKQEEMS(ph)PR (A), EKY(ph)DKLASPEDSASETTMATQPQK (B), YDKLAS(ph)PEDSASETTMATQPQK (C), YDKLASPEDSAS(ph)ETTMATQPQK (D), VAEHS(ph)SAAVTDR/VAEHSS(ph)AAVTDR (E), VAEHSSAAVT(ph)DR (F), MLC1 phosphopeptides VGEYDGACES(ph)PSCR (G), VST(ph)GDAMILAR (H), SGDNLDYAS(ph)FQK (I), or MyoA phosphopeptide RSS(ph)DVHAVDHSGNVYK (I) listed in Table 1. The peptide sequences for phosphoptptides (top left), observed mass (AMU), parent error (ppm), and MS/MS spectra (relative intensity vs. m/z) of the [M+2H]^2+^ or [M+3H]^3+^ precursor are shown: Matching b-ions (red) and y-ions (blue) with consecutive neutral losses of phosphoric acid allow an unambiguous localization in most cases. Prominent parent ion peaks showing neutral loss of phosphoric acid are also shown.
(TIF)

Figure S6 **Quantification and localization of Ca^2+^-dependent** ***T. gondii*** **GAP45 phosphorylation sites listed in** **Table 1** **(manual validation).** A) 2D gel electrophoresis reveals the presence of three additional phosphorylated GAP45 species in ethanol-treated *Toxoplasma* tachyzoite lysates. Only two ^32^[P]-labeled species were detected in anti-GAP45 immunoprecipitates from untreated parasites (-), whereas samples prepared in parallel from ethanol-treated parasites (+) show five radioactive 2D spots with different isoelectric points (tick marks). This demonstrates the presence of three Ca^2+^-dependent phosphorylation sites on GAP45. Samples were immunoprecipitated from a Triton X-100 lysate of ^32^[P] orthophosphate-labeled tachyzoites using polyclonal anti-GAP45 rabbit antiserum, separated by 2-DE, transferred onto PVDF membranes and autoradiographed, as detailed in Materials & Methods. The acidic and basic ends of the IEF strips are indicated. B) Extracted ion chromatograms (XICs) for m/z 661.79 and m/z 663.79 representing the [M+2H]^2+^ ion of the light (R0)- (gray) or heavy (R+4)-labeled (black) VAEHSSAVT(ph)DR (T189) or VAEHS(ph)SAVTDR/VAEHSS(ph)AVTDR (S184/5) phosphopeptides. The elution profile shows three peaks at RT 38.43, RT 38.72, as indicated. C) The relative intensity of the light (m/z 661.79) and heavy (m/z 663.79) [M+2H]^2+^ ions at RT 38.38 - 38.48 (peak 1) or RT 38.72 – 88.85 (peak 2 and 3) are shown, in accordance with normalized data shown in Table 1. D) Annotated MS/MS fragmentation spectra of the [M+2H]^2+^ ion at RT 38.39 (peak 1) or the [M+3H]^3+^ ion at RT 38.87 (peak 2/3) representing the heavy (R+4)-labeled phosphopeptide VAEHSSAVT(ph)DR (top). The chimera MS/MS spectrum at the bottom probably represents a mixture of VAEHS(ph)SAVTDR/VAEHSS(ph)AVTDR parent ions (S184/5), as indicated by the presence of both corresponding phosphorylated (b+80) as well as neutral loss (y7–98) fragment ions (arrows). Please, refer to Supplementary Text S2 for a detailed discussion of these results.
(TIF)

Figure S7 **Quantification and localization of Ca^2+^-dependent MLC1 phosphorylation sites listed in** **Table 1** **(manual validation).** A) Extracted ion chromatograms for SILAC pairs m/z 833.80/635.79 (S53), m/z 607.29/609.28 (T98), or m/z 712.79/716.80 (S132) representing the [M+2H]^2+^ ions of light (R0)- (gray) and heavy (R+4 or K+8)-labeled (black) monophosphorylated peptides VGEYDGAcES(ph)PScR, VSTGDAMILAR, or SGDNLDYASFQK, as indicated. **B)** The relative intensity of the light and heavy [M+2H]^2+^ ions for the phosphopeptide peaks at RT 41.48 (S53), RT 49.57 (T98), or 48.80 (S132) are shown, in accordance with normalized data shown in Table 1. **C)** Annotated MS/MS fragmentation spectra of the [M+2H]^2+^ ions representing the phosphopeptide sequences VGEYDGAcES(ph)PScR, VSTGDAMILAR, or SGDNLDYASFQK. Neutral-loss b-98 or y-98 fragment ions are indicated. Please, refer to Supplementary Text S3 for a detailed discussion of these results.
(TIF)

Figure S8 **LC-MS/MS identification of ELC1 by anti-HA CoIP from a MLC-HA expressing parasite line (15** **kDa band shown in** **Figure 4E** **).** Sequence coverage for *Toxoplasma* calmodulin-like protein TGME49_069440 (ELC1) determined by LC-MS/MS analysis of a Sypro Ruby-stained 15-kDa protein band detected in anti-HA column eluates of MLC1-HA expressing parasites, as shown in Figure 4E. Database accession numbers (ToxoDB v5.0 IDs) and protein name are shown. Identified peptide sequences are highlighted in yellow and modified or labeled residues are shown in green.
(TIF)

Figure S9 **Sequence alignment of MyoA N- and C-terminal domains.** ClustalW alignment of the conserved N-terminal sequences (top) and C-terminal tails (bottom) of MyoA orthologues from (top to bottom) *P. falciparum* (PF13_0233), *P. knowlesi* (PKH_121190), *P. vivax* (PVX_083030), *P. chabaudi* (PCAS_136030*), P. yoelii* (PY01232), *Neospora caninum* (NCLIV_049900) and *T. gondii* (TGME49_035470). The Ca^2+^-dependent MLC1 phosphorylation S21 (Ca^2+^↑) is shown. C-terminal residues involved in interactions with MTIP [33] or necessary for peripheral localization of MyoA are also indicated above or below the alignment (asterisks). The position of the proposed ELC1 and MLC1 binding regions based on sequence and structural similarity with the regulatory domain of scallop myosin is indicated by bars. Residues conserved in all 7 apicomplexan (class XIV) myosins are highlighted in red and all potential S/T/Y phosphorylation sites are highlighted in yellow.
(TIF)

Figure S10 **Sequence alignment of** ***Toxoplasma*** **MyoA, ELC1 and MLC1 with the structure of the complex of Myosin II, the Essential Light Chain and the Regulatory Light Chain in** ***Physarum polycephalum.*** Sequence alignment of *Toxoplasma* MyoA, ELC1 and MLC1 with the structure of the complex of Myosin II, the Essential Light Chain and the Regulatory Light Chain in *Physarum polycephalum* (PDBid 2BL0). Protein fold recognition was conducted using the WURST protein-threading web server [52]. Homology models were constructed using the sequence alignments predicted from WURST with the MODELLER (9v7) comparative modeling software [53]. The sequences of *T. gondii* MLC1 and ELC1 were identified to be highly compatible with the structure of the essential light chain of the *Physarum* myosin II complex (PDB 2BL0, chains C and B, respectively). Despite the high level of sequence similarity between MLC1 proteins presented in Figure 6B, few of these residues participate in direct interactions with the MyoA tail, and all residues that do interact with MyoA are hydrophobic in nature (Figure 6B, asterisks), including L108 and L110 in the N-lobe, and L150, F154, L174, L181 and F204 in the C-lobe (see Figure 6C). The model of *T. gondii* ELC1 shown in Figure 4G includes a calcium binding site juxtaposed near the region of closest approach between the ELC1 (residues T16-D17) and MLC1 (residues Y177-G178-E179) – homologues of G178 are highly conserved amongst MLC1s and other calmodulins.
(TIF)

Table S1 **Summary of 2-DE-based LC-MS/MS analyses.** Fifty ^32^[P]-labelled parasite protein spots were matched, excised from preparative 2D gel electrophoresis and subjected to LC-MS/MS analysis. Listed, from left to right: Protein name, ToxoDB release 6.2 ID (Accession #), theoretical molecular mass (Mr), protein identification probability (%), Joint Proteomics Service Facility project number (JPSF #) and 2-DE reference number (#1-50) for ^32^[P]-labelled protein spots shown in Figure S1. Phosphorylated proteins identified by MudPIT are indicated in column 1 and highlighted in red.
(XLS)

Table S2 **Summary of Sequest-based database searches of MudPIT data (TiO_2_ unbound).** Whole *Toxoplasma* protein extract was digested with trypsin, fractionated using hydrophilic interaction chromatography and the resulting 10 fractions partitioned using TiO_2_ affinity chromatography. The resulting TiO_2_-unbound fractions were combined and subjected to MudPIT analysis using a 6-step gradient and searched against a non-redundant *Toxoplasma* decoy database using the Sequest search engine, as detailed in Materials and Methods. Listed from left to right (top row; grey): Locus (ToxoDB v5.0 database accession number), Descriptive name (Protein name in the ToxoDB v5.0 database), Spectrum Count (total number of peptides identified for this protein); % Coverage (percent Protein sequence coverage); Length (protein length in amino acid residues); MolWt (Molecular weight of the protein); pI (pI of protein). Listed from left to right (bottom rows; white): Unique (*: indicates this is a unique peptide sequence in the protein database used), Xcorr (SEQUEST Xcorr, Cross-correlation score), DeltCN (SEQUEST Delta CN score); Con% (peptide ID confidence), M+H^+^ (observed molecular weight of the protonated peptide), Calc M+H^+^ (calculated molecular weight of the protonated peptide), Prob Score (Prolucid Z Score; a Zscore of 5 means that the top hit is 5 standard deviations away from average), Sequence (peptide sequence).
(XLS)

Table S3 **Summary of Sequest-based database searches of MudPIT data (TiO_2_ bound).** Whole *Toxoplasma* protein extract was digested with trypsin, fractionated using hydrophilic interaction chromatography and the resulting 10 fractions partitioned using TiO_2_ affinity chromatography. The resulting TiO_2_-bound fractions were individually and subjected to MudPIT analysis using a 2-step gradient and searched against a non-redundant *Toxoplasma* decoy database using the Sequest search engine, as detailed in Materials and Methods. Listed from left to right (top row; grey): Locus (ToxoDB v5.0 database accession number), Descriptive name (Protein name in the ToxoDB v5.0 database), Spectrum Count (total number of peptides identified for this protein); % Coverage (percent Protein sequence coverage); Length (protein length in amino acid residues); MolWt (Molecular weight of the protein); pI (pI of protein). Listed from left to right (bottow row; white): Unique (*: indicates this is a unique peptide sequence in the protein database used), Xcorr (SEQUEST Xcorr, Cross-correlation score), DeltCN (SEQUEST Delta CN score); Con% (peptide ID confidence), M+H^+^ (observed molecular weight of the protonated peptide), Calc M+H^+^ (calculated molecular weight of the protonated peptide), Prob Score (Prolucid Z Score; a Zscore of 5 means that the top hit is 5 standard deviations away from average), Sequence (peptide sequence), Debunker Score (Debunker Phosphopeptide ID Score).
(XLS)

Table S4 **Summary of MASCOT-based database searches of MudPIT data (TiO_2_ bound).** Whole *Toxoplasma* protein extract was digested with trypsin, fractionated using hydrophilic interaction chromatography and the resulting 10 fractions partitioned using TiO_2_ affinity chromatography. The resulting TiO_2_-bound fractions were individually and subjected to MudPIT analysis using a 2-step gradient and searched against a custom *Toxoplasma* decoy database using the Mascot search engine, as detailed in Materials and Methods. Listed from left to right: Protein name (Protein name in the database used) Accession No. (Tremble, SwissProt, UniProt or ToxoDB v6.2 accession numbers in the database used), Prot ID Prob (protein ID confidence in %), Pep ID Prob (peptide ID confidence in %), # Spectra (total number of spectra identified for this peptide); % coverage (percent Protein sequence coverage of this peptide); m/z (observed mass over charge of this peptide); AMU actual (observed molecular weight of this peptide in atomic mass units) AMU calc (calculated molecular weight of this peptide in atomic mass units), Mascot Score (only highly confident peptide IDs with a Mascot Score cutoff of >30 are shown), Phosphopeptide sequence (sequence of the identified phosphopeptide).
(XLS)

Table S5 **Phosphopeptide identification and quantification of anti-GAP45 column eluates (MaxQuant Report).** Listed from left to right: Protein/Protein Description (accession number and protein name listed in the Toxoplasma_decoy database used), aa (phosphorylated S/T/Y residue), Pos (position of phosphorylated residue in the protein sequence), LocProb (MaxQuant phosphorylation site localization probability), Modified Sequence (sequence of identified phosphopeptide), Phos (STY) Probabilities (MaxQuant phosphorylation site localization probabilities for different STY phosphorylation sites in the sequence), Mascot Score (Mascot score for best localization spectrum identified by MaxQuant), Ratio H/L Count (average H:L Ratio of heavy and light SILAC pairs for this phosphopeptide), Ratio H/L Count Normalized by Protein (average H:L Ratio of heavy and light SILAC pairs for this phosphopeptide divided by the average H:L Ratio for all SILAC pairs detected for the whole protein), Manual validation (result of manual annotation of LC-MS/MS evidence spectra and inspection of XICs of associated SILAC pairs). For a more detailed interpretation of the result for GAP45 phosphorylation sites S184 and S185 (asterisks) please refer to Supplementary Figure S6 and Text S2.
(XLS)

Table S6 **Component analysis of intact invasion motor complexes by LC-MS/MS.** Listed from left to right: Rank (ranking based on the total number of confident IDs (<1%FDR) and combined total number spectra from 8 independent anti-GAP45 immunoprecipitation experiments). # IDs (number of confident IDs (<1%FDR) out of 8 experiments), Spectrum count (combined total number spectra from 8 independent experiments), Protein description (accession number and protein name listed in the Toxoplasma_decoy database used), Mr (Molecular weight of the protein). Results have been filtered to remove highly abundant *Toxoplasma* proteins commonly identified in IPs with beads coated with non-specific rabbit IgG. Typical ‘contaminants’ included ribosomal subunits, heat-shock proteins, histones, or dense granule proteins (not shown). Proteins consistently identified in >4 independent experiments are highlighted in red.
(XLS)

Table S7 **Protein quantification of anti-GAP45 column eluates (MaxQuant report).** Listed from left to right: Protein description (accession number and protein name listed in the Toxoplasma_decoy database used), Mr (Molecular weight of the protein), Seq Cov [%] (percent Protein sequence coverage), Peptide # (total number of peptides identified for this protein), H/L Count (number of identified SILAC pairs for this protein), Intensity L (Intensity of the light ion), Intensity H (Intensity of the heavy ion), H/L ratio (ratio of heavy to light ions calculated by the MaxQuant ‘Quant’ module, D  =  Disorder).
(XLS)

Table S8 **Prediction of disordered regions of the MLC1 N-terminal domain.** Listed from left to right: AA (residue number), seq (amino acid residue), disembl/poodlel/iupreds/iupredl/poodles/prdos/ronn/globplot/disprot (result of different disorder predictors along the N-terminal 80 aa of MLC1), Concensus (concensus of all 9 predictors along the N-terminal 80 aa of MLC1, D  =  disorder).
(XLS)

Text S1 **MS database searches of MudPIT spectra, phosphoprotein identification and statistical analysis.** Summary and statistics of phosphopeptide identifications based on Sequest or Mascot database searches of MudPIT raw data, as listed in Supplementary Tables S3 and S4
(DOC)

Text S2 **Ca^2+^-dependent phosphorylation of GAP45.** Interpretation of SILAC-based quantitative MS data for Ca^2+^-dependent phosphorylation sites of GAP45, as shown in Supplementary Figure S6.
(DOC)

Text S3 **Ca^2+^-dependent phosphorylation of MLC1.** Interpretation of SILAC-based quantitative MS data for Ca^2+^-dependent phosphorylation sites of MLC1, as shown in Supplementary Figure S7.
(DOC)
