# Supplementary figures and images for: Quantitative in vivo Analyses Reveal Calcium-dependent Phosphorylation Sites and Identifies a Novel Component of the Toxoplasma Invasion Motor Complex
Source: PLoS Pathog. 2011 Sep 29;7(9):e1002222. doi: 10.1371/journal.ppat.1002222 (PMC3182922; doi:10.1371/journal.ppat.1002222)

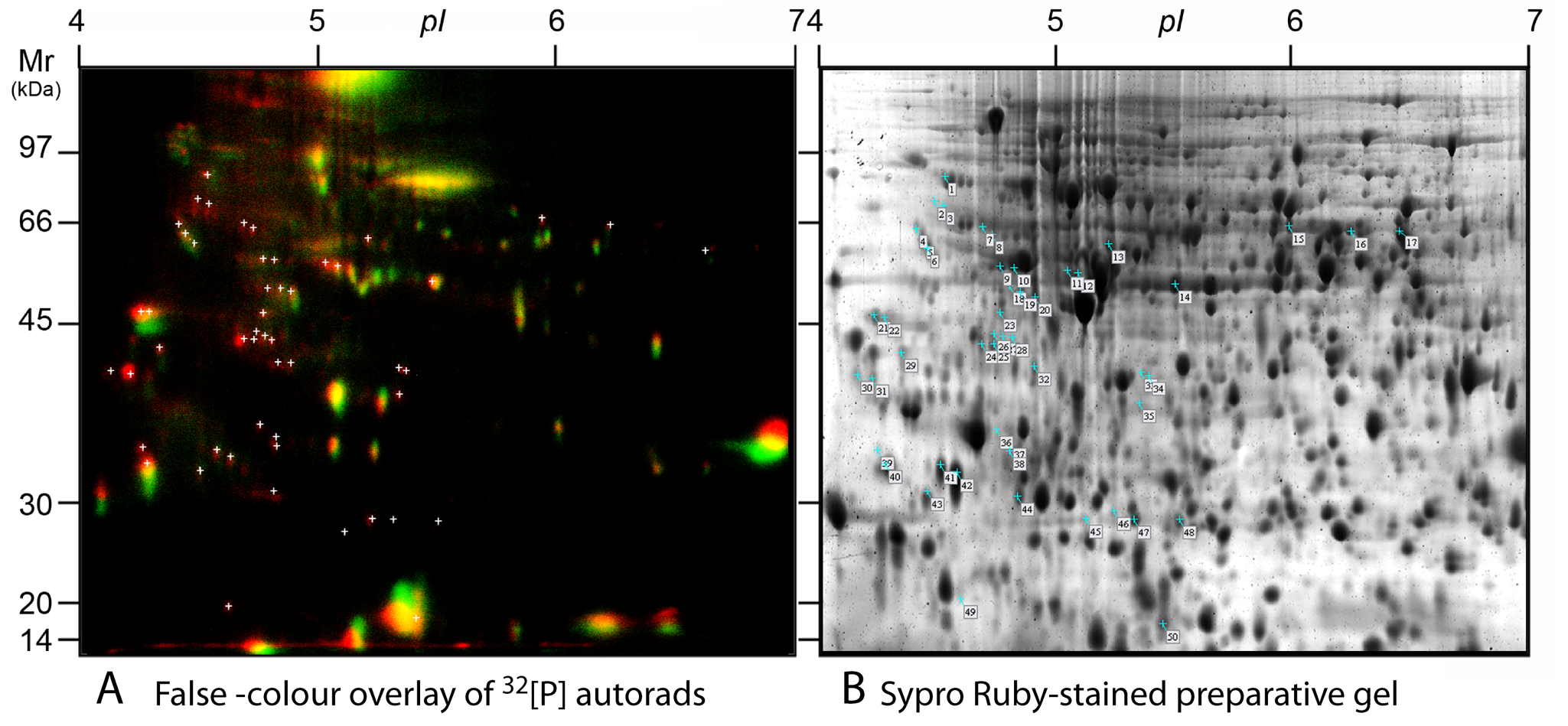

Supplement: Figure S1 — Matching of 32[P]-labeled 2-DE spots for the LC-MS/MS based identification of Ca2+-dependent Toxoplasma proteins listed in Table S1. A) For 2-DE-based identification of phosphorylated proteins upon Ca2+ pathway stimulation 32[P]-orthophosphate labeled parasites were treated with (red) or without (green) ionomycin. Phosphoproteins were detected via autoradiography and fake coloured for comparison, as detailed in Materials and Methods. B) For the MS-based identification of Toxoplasma phosphoproteins, 500 µg aliquots of total parasite protein extract were separated on preparative 2D gels and imaged using Sypro Ruby protein stain (Molecular Probes). Fifty parasite phosphoprotein spots that were consistently labeled following Ca2+ pathway stimulation were matched, corresponding regions manually excised from preparative 2D gel electrophoresis and subjected to nanoLC-MS/MS analysis, as detailed in Materials and Methods. Please, refer to Supplementary Table S1 for corresponding protein identification results. (TIF) [file ppat.1002222.s001.tif]

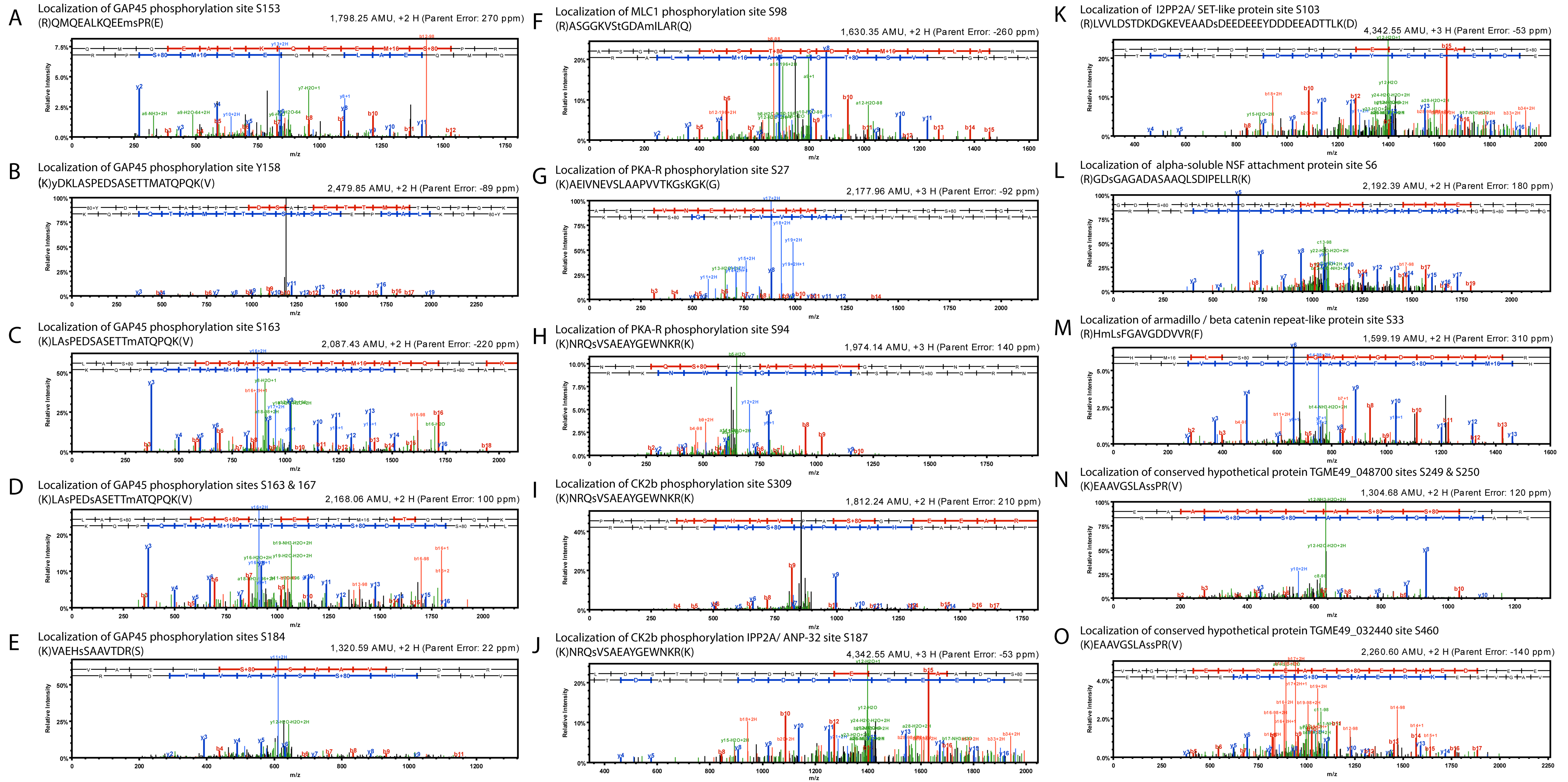

Supplement: Figure S2 — MudPIT (LTQ) MS/MS evidence spectra for phosphopeptides listed in Figure 2B . Phosphorylation site localization for the phosphopeptides listed in Table 1. A. GAP45 p153, B. GAP45, pY158 C. GAP45 pS163, D. GAP45 pS163 & pS167, E. GAP45 pS184, F. MLC1 S98, G. PKA-R pS27, H. PKA-R pS94, I. CKIIβ pS309, J. IPP2A/ANP-32 pS187, K. I2PP2A/SET-like pS103, L. SNAP pS6, M. ARM1 pS33, N. Hyp TGME49_048700 pS249 & pS250, O. Hyp TGME49_032440 pS460. The peptide sequences (top left), observed mass (AMU), parent error (ppm), and central regions of the MS/MS spectra (relative intensity vs. m/z) of the [M+2H]2+ or [M+3H]3+ precursor are shown: Matching b-ions (red) and y-ions (blue) with consecutive neutral losses of phosphoric acid allow a clear-cut annotation in most cases. Prominent parent ion peaks showing neutral loss of phosphoric acid are not shown. (TIF) [file ppat.1002222.s002.tif]

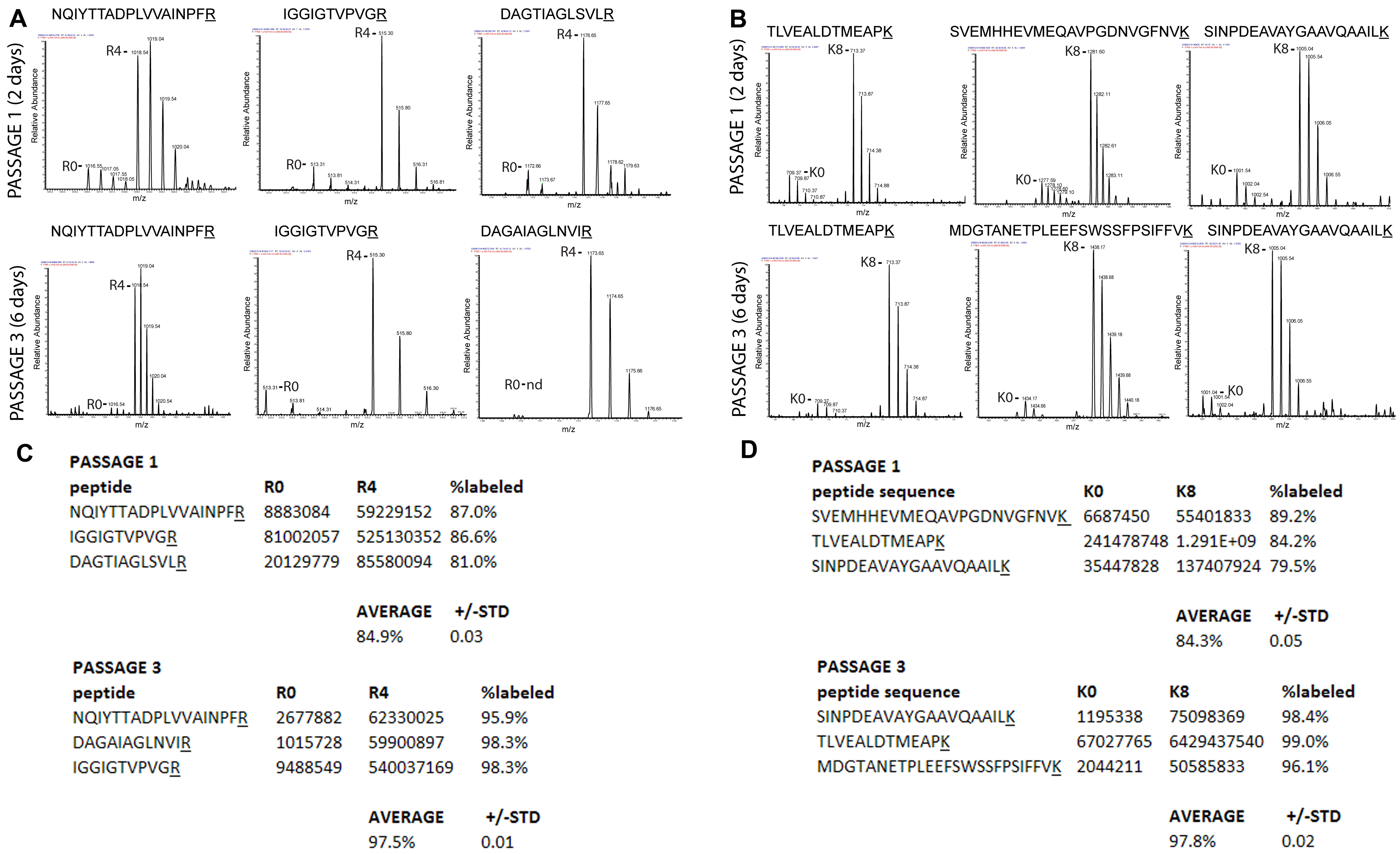

Supplement: Figure S3 — Quantitative analysis of [15N4] L-Arginine (R4) or [13C6, 15N2] L-Lysine (K8) incorporation in Toxoplasma. For the analysis of [15N4] L-Arginine incorporation, Toxoplasma parasites were grown in ‘heavy’ [15N4] L-Arginine (R4) or [13C6, 15N2] L-Lysine (K8) SILAC labeling media for 1 or 3 passages, as detailed in Materials & Methods. Heavy label incorporation was applied for up to 6 days in HFF cells before parasite harvest, as indicated. Parasite protein was extracted, digested in solution and analyzed using nano-LC-MS/MS on an LTQ-OrbiTrap instrument. MS data were searched against a Toxoplasma_decoy database using the MASCOT search engine, and the Xcalibur program (Thermo Scientific) was used to plot the relative abundance of ‘light (R0) versus ‘heavy’ (R4) arginine-labeled peptides (A), or ‘light’ (K0) versus heavy (K8) lysine-labled peptides (B). A statistical summary of SILAC label incorporation can be found in panels C and D. (TIF) [file ppat.1002222.s003.tif]

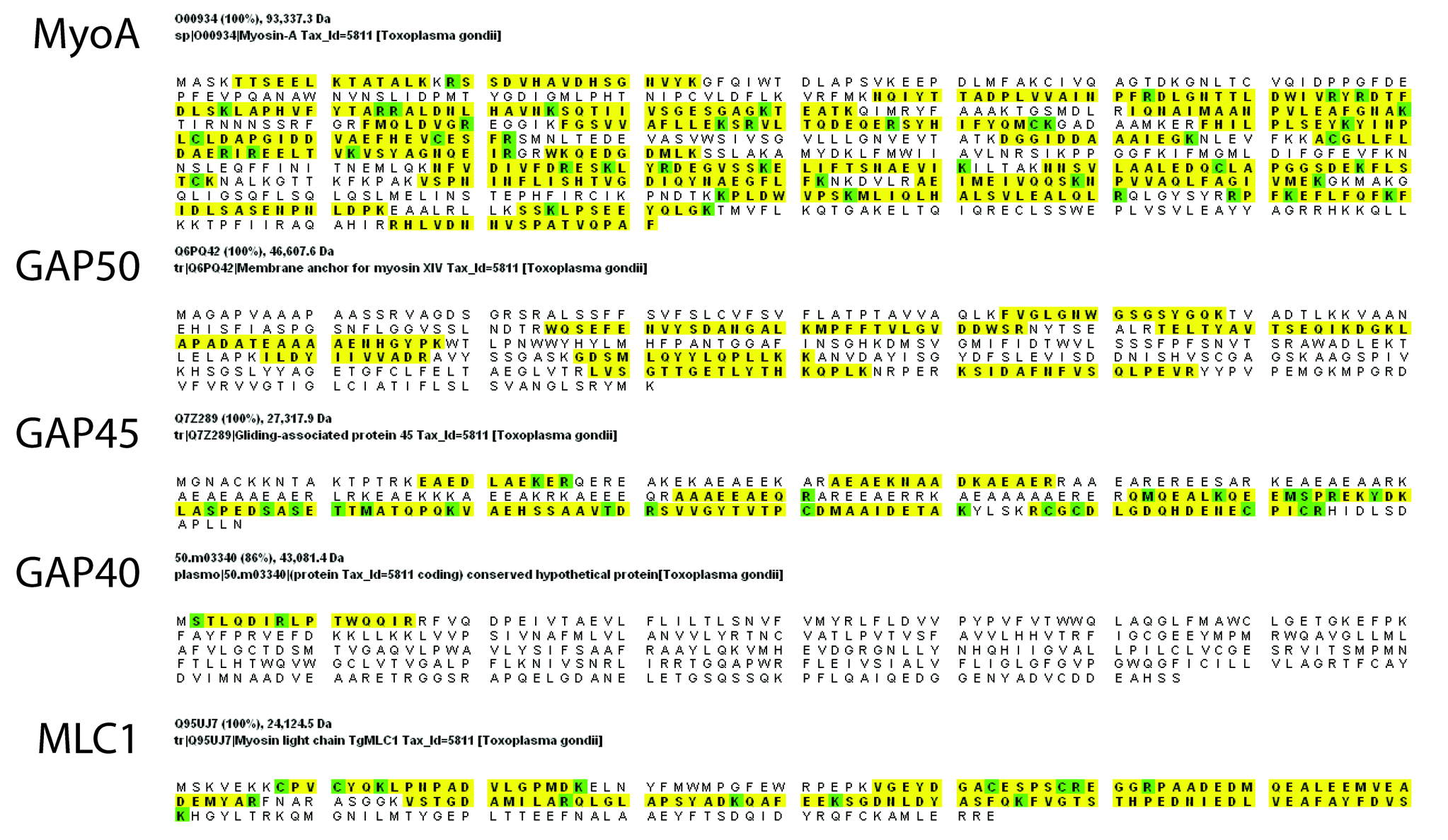

Supplement: Figure S4 — Sequence coverage of invasion motor components. Sequence coverage for MyoA, GAP50, GAP45, GAP40, MLC1 peptides determined by LC-MS/MS analysis of tryptic digest of intact Toxoplasma invasion motor complex components (anti-GAP45 column eluates), as shown in Figure 3B. Database accession numbers (ToxoDB v5.0 IDs) and protein name are shown. Identified peptide sequences are highlighted in yellow and modified or labeled residues are shown in green. (TIF) [file ppat.1002222.s004.tif]

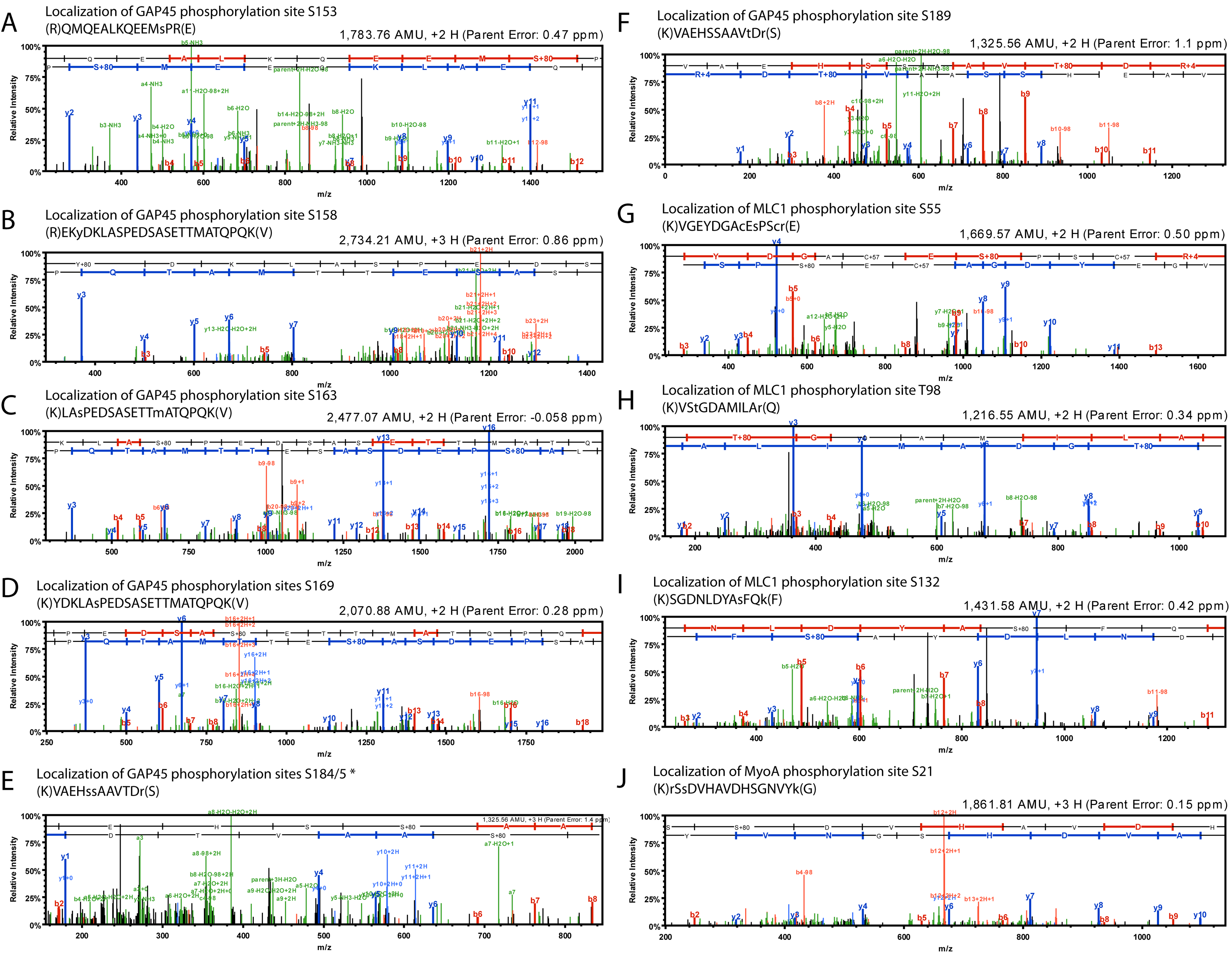

Supplement: Figure S5 — Orbitrap MS/MS evidence spectra for phosphopeptides listed in Table 1 . Phosphorylation site localization for the GAP45 phosphopeptides QMQEALKQEEMS(ph)PR (A), EKY(ph)DKLASPEDSASETTMATQPQK (B), YDKLAS(ph)PEDSASETTMATQPQK (C), YDKLASPEDSAS(ph)ETTMATQPQK (D), VAEHS(ph)SAAVTDR/VAEHSS(ph)AAVTDR (E), VAEHSSAAVT(ph)DR (F), MLC1 phosphopeptides VGEYDGACES(ph)PSCR (G), VST(ph)GDAMILAR (H), SGDNLDYAS(ph)FQK (I), or MyoA phosphopeptide RSS(ph)DVHAVDHSGNVYK (I) listed in Table 1. The peptide sequences for phosphoptptides (top left), observed mass (AMU), parent error (ppm), and MS/MS spectra (relative intensity vs. m/z) of the [M+2H]2+ or [M+3H]3+ precursor are shown: Matching b-ions (red) and y-ions (blue) with consecutive neutral losses of phosphoric acid allow an unambiguous localization in most cases. Prominent parent ion peaks showing neutral loss of phosphoric acid are also shown. (TIF) [file ppat.1002222.s005.tif]

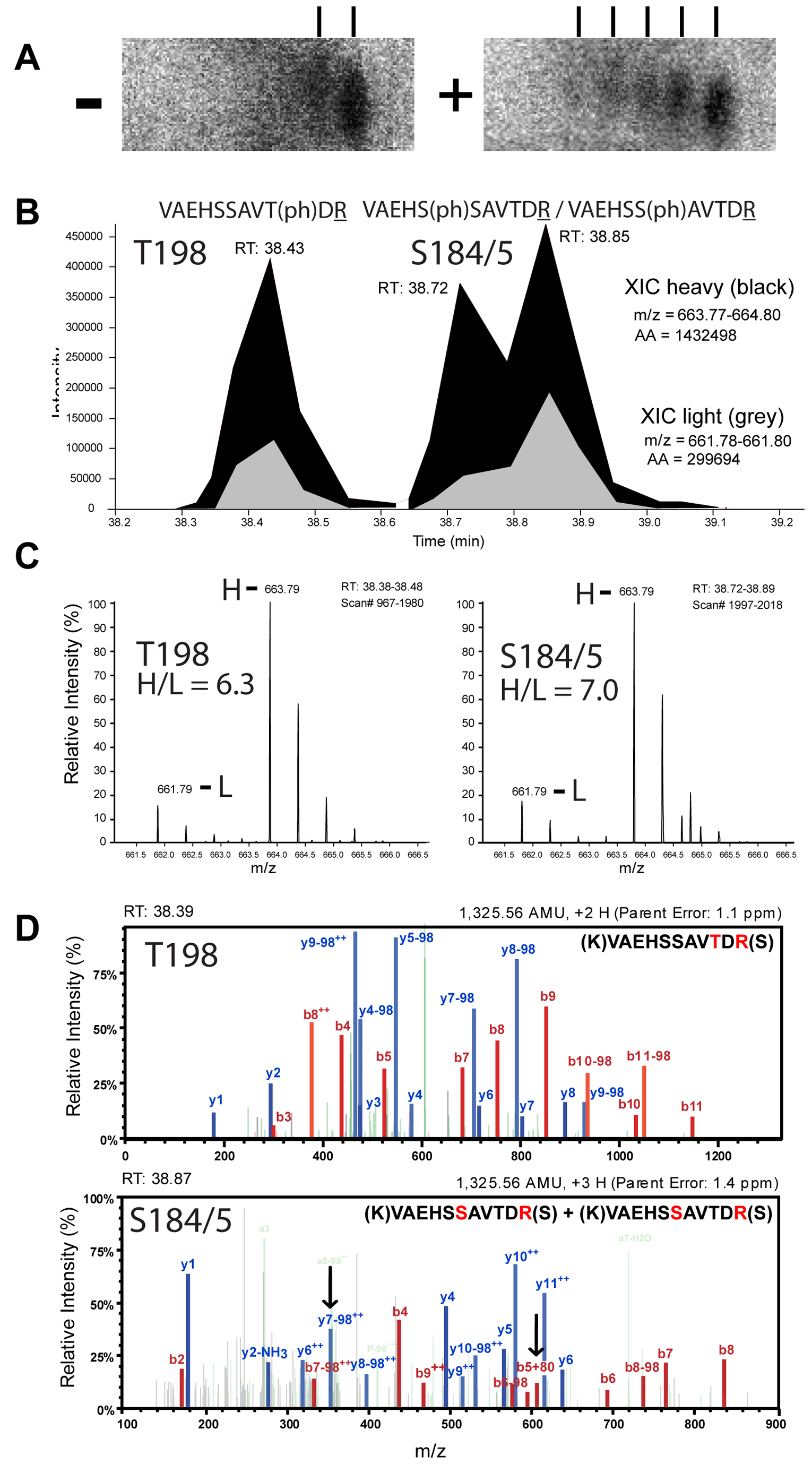

Supplement: Figure S6 — Quantification and localization of Ca2+-dependent T. gondii GAP45 phosphorylation sites listed in Table 1 (manual validation). A) 2D gel electrophoresis reveals the presence of three additional phosphorylated GAP45 species in ethanol-treated Toxoplasma tachyzoite lysates. Only two 32[P]-labeled species were detected in anti-GAP45 immunoprecipitates from untreated parasites (-), whereas samples prepared in parallel from ethanol-treated parasites (+) show five radioactive 2D spots with different isoelectric points (tick marks). This demonstrates the presence of three Ca2+-dependent phosphorylation sites on GAP45. Samples were immunoprecipitated from a Triton X-100 lysate of 32[P] orthophosphate-labeled tachyzoites using polyclonal anti-GAP45 rabbit antiserum, separated by 2-DE, transferred onto PVDF membranes and autoradiographed, as detailed in Materials & Methods. The acidic and basic ends of the IEF strips are indicated. B) Extracted ion chromatograms (XICs) for m/z 661.79 and m/z 663.79 representing the [M+2H]2+ ion of the light (R0)- (gray) or heavy (R+4)-labeled (black) VAEHSSAVT(ph)DR (T189) or VAEHS(ph)SAVTDR/VAEHSS(ph)AVTDR (S184/5) phosphopeptides. The elution profile shows three peaks at RT 38.43, RT 38.72, as indicated. C) The relative intensity of the light (m/z 661.79) and heavy (m/z 663.79) [M+2H]2+ ions at RT 38.38 - 38.48 (peak 1) or RT 38.72 – 88.85 (peak 2 and 3) are shown, in accordance with normalized data shown in Table 1. D) Annotated MS/MS fragmentation spectra of the [M+2H]2+ ion at RT 38.39 (peak 1) or the [M+3H]3+ ion at RT 38.87 (peak 2/3) representing the heavy (R+4)-labeled phosphopeptide VAEHSSAVT(ph)DR (top). The chimera MS/MS spectrum at the bottom probably represents a mixture of VAEHS(ph)SAVTDR/VAEHSS(ph)AVTDR parent ions (S184/5), as indicated by the presence of both corresponding phosphorylated (b+80) as well as neutral loss (y7–98) fragment ions (arrows). Please, refer to Supplementary Text S2 for a detailed discussion of these re [file ppat.1002222.s006.tif]

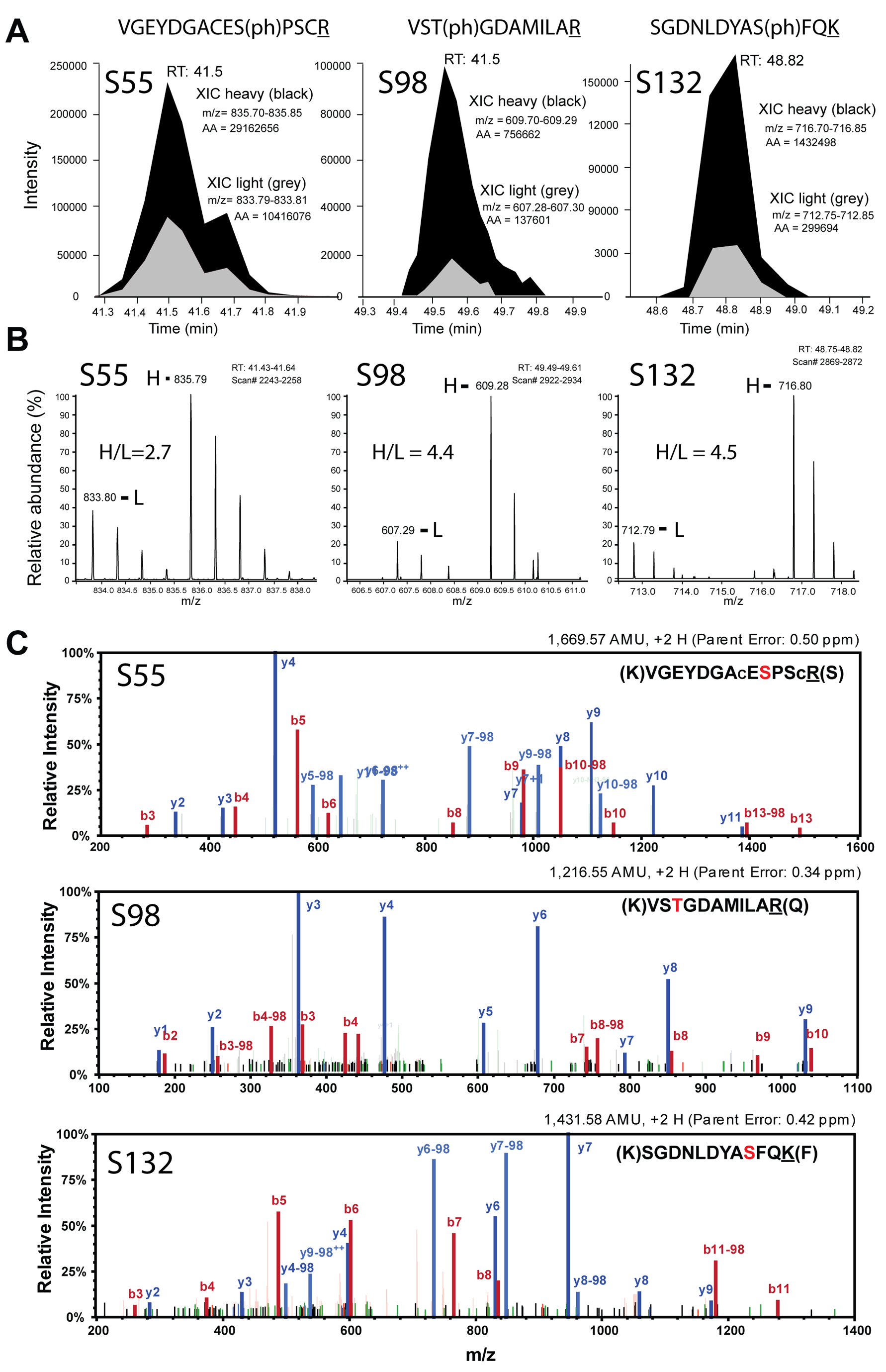

Supplement: Figure S7 — Quantification and localization of Ca2+-dependent MLC1 phosphorylation sites listed in Table 1 (manual validation). A) Extracted ion chromatograms for SILAC pairs m/z 833.80/635.79 (S53), m/z 607.29/609.28 (T98), or m/z 712.79/716.80 (S132) representing the [M+2H]2+ ions of light (R0)- (gray) and heavy (R+4 or K+8)-labeled (black) monophosphorylated peptides VGEYDGAcES(ph)PScR, VSTGDAMILAR, or SGDNLDYASFQK, as indicated. B) The relative intensity of the light and heavy [M+2H]2+ ions for the phosphopeptide peaks at RT 41.48 (S53), RT 49.57 (T98), or 48.80 (S132) are shown, in accordance with normalized data shown in Table 1. C) Annotated MS/MS fragmentation spectra of the [M+2H]2+ ions representing the phosphopeptide sequences VGEYDGAcES(ph)PScR, VSTGDAMILAR, or SGDNLDYASFQK. Neutral-loss b-98 or y-98 fragment ions are indicated. Please, refer to Supplementary Text S3 for a detailed discussion of these results. (TIF) [file ppat.1002222.s007.tif]

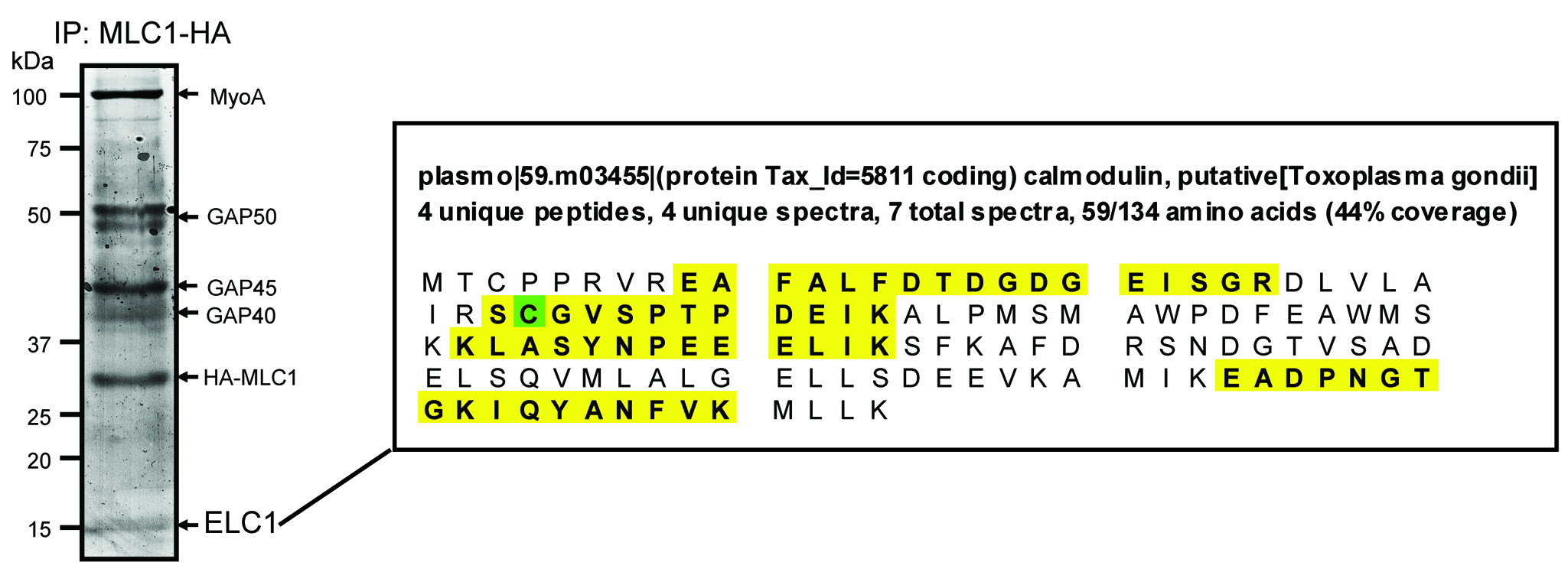

Supplement: Figure S8 — LC-MS/MS identification of ELC1 by anti-HA CoIP from a MLC-HA expressing parasite line (15 kDa band shown in Figure 4E ). Sequence coverage for Toxoplasma calmodulin-like protein TGME49_069440 (ELC1) determined by LC-MS/MS analysis of a Sypro Ruby-stained 15-kDa protein band detected in anti-HA column eluates of MLC1-HA expressing parasites, as shown in Figure 4E. Database accession numbers (ToxoDB v5.0 IDs) and protein name are shown. Identified peptide sequences are highlighted in yellow and modified or labeled residues are shown in green. (TIF) [file ppat.1002222.s008.tif]

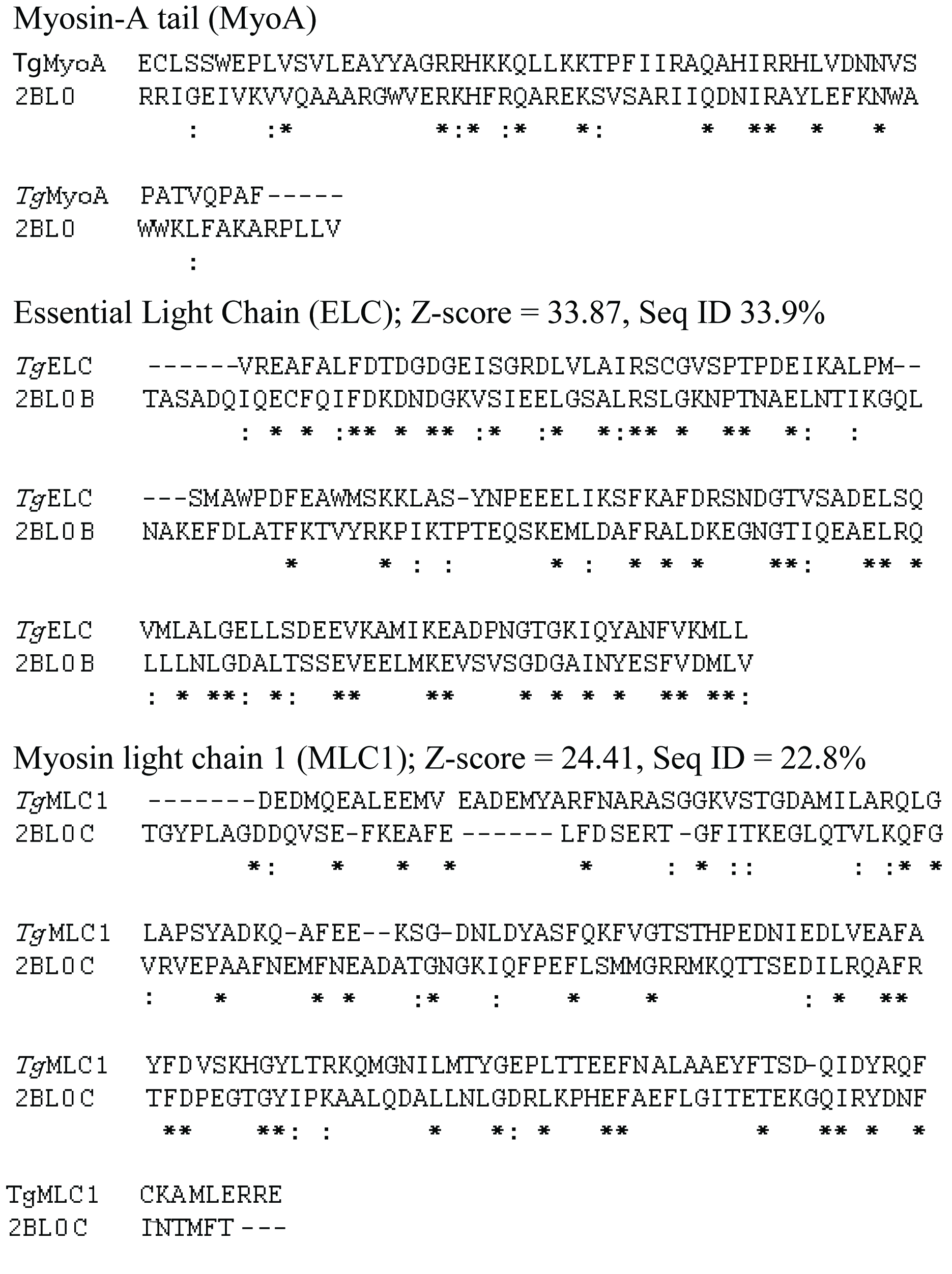

Supplement: Figure S9 — Sequence alignment of MyoA N- and C-terminal domains. ClustalW alignment of the conserved N-terminal sequences (top) and C-terminal tails (bottom) of MyoA orthologues from (top to bottom) P. falciparum (PF13_0233), P. knowlesi (PKH_121190), P. vivax (PVX_083030), P. chabaudi (PCAS_136030), P. yoelii (PY01232), Neospora caninum (NCLIV_049900) and T. gondii (TGME49_035470). The Ca2+-dependent MLC1 phosphorylation S21 (Ca2+↑) is shown. C-terminal residues involved in interactions with MTIP [33] or necessary for peripheral localization of MyoA are also indicated above or below the alignment (asterisks). The position of the proposed ELC1 and MLC1 binding regions based on sequence and structural similarity with the regulatory domain of scallop myosin is indicated by bars. Residues conserved in all 7 apicomplexan (class XIV) myosins are highlighted in red and all potential S/T/Y phosphorylation sites are highlighted in yellow. (TIF) [file ppat.1002222.s009.tif]

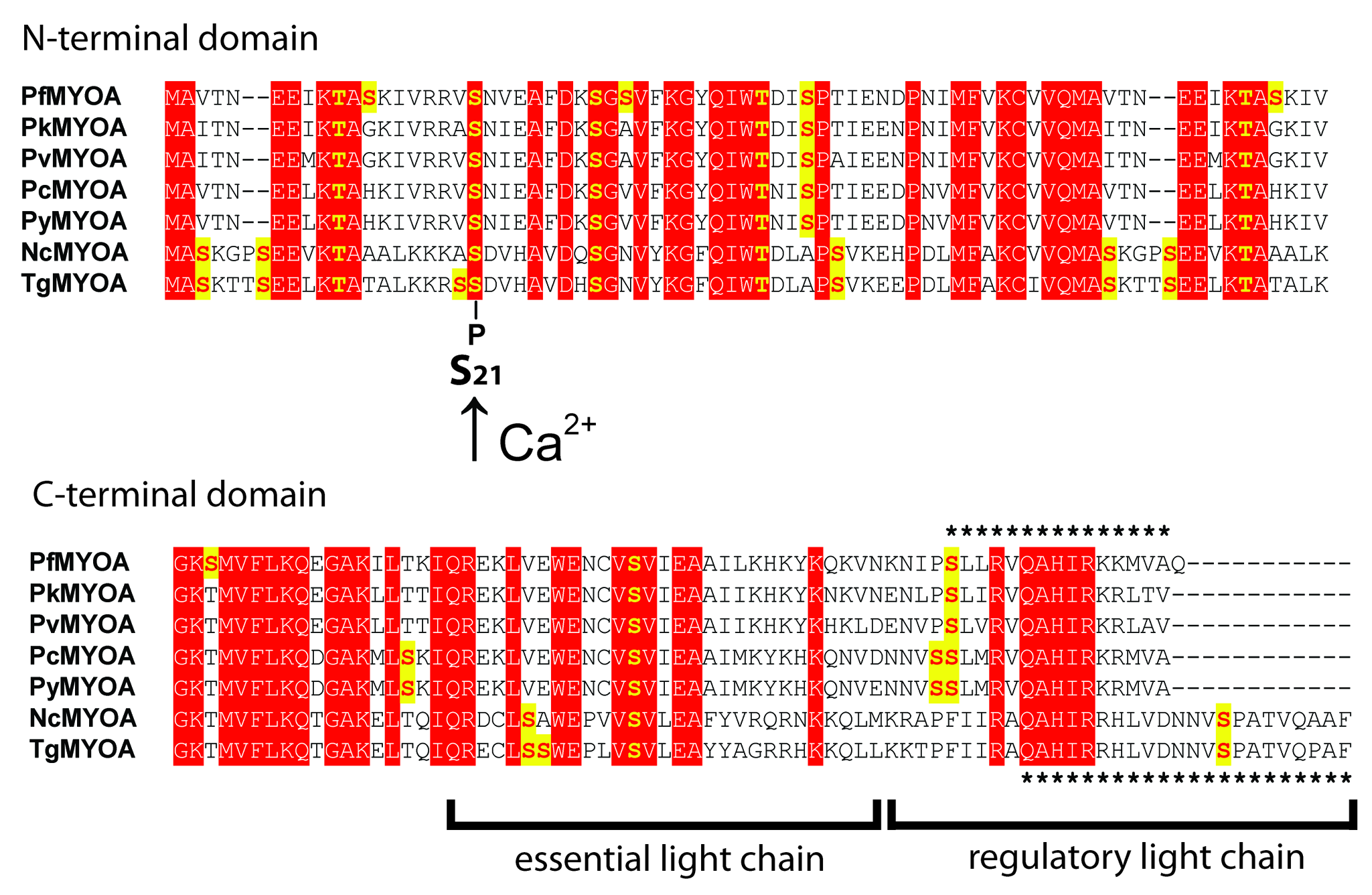

Supplement: Figure S10 — Sequence alignment of Toxoplasma MyoA, ELC1 and MLC1 with the structure of the complex of Myosin II, the Essential Light Chain and the Regulatory Light Chain in Physarum polycephalum. Sequence alignment of Toxoplasma MyoA, ELC1 and MLC1 with the structure of the complex of Myosin II, the Essential Light Chain and the Regulatory Light Chain in Physarum polycephalum (PDBid 2BL0). Protein fold recognition was conducted using the WURST protein-threading web server [52]. Homology models were constructed using the sequence alignments predicted from WURST with the MODELLER (9v7) comparative modeling software [53]. The sequences of T. gondii MLC1 and ELC1 were identified to be highly compatible with the structure of the essential light chain of the Physarum myosin II complex (PDB 2BL0, chains C and B, respectively). Despite the high level of sequence similarity between MLC1 proteins presented in Figure 6B, few of these residues participate in direct interactions with the MyoA tail, and all residues that do interact with MyoA are hydrophobic in nature (Figure 6B, asterisks), including L108 and L110 in the N-lobe, and L150, F154, L174, L181 and F204 in the C-lobe (see Figure 6C). The model of T. gondii ELC1 shown in Figure 4G includes a calcium binding site juxtaposed near the region of closest approach between the ELC1 (residues T16-D17) and MLC1 (residues Y177-G178-E179) – homologues of G178 are highly conserved amongst MLC1s and other calmodulins. (TIF) [file ppat.1002222.s010.tif]
