# Supplementary material for: Quantitative in vivo Analyses Reveal Calcium-dependent Phosphorylation Sites and Identifies a Novel Component of the Toxoplasma Invasion Motor Complex
Source: PLoS Pathog. 2011 Sep 29;7(9):e1002222. doi: 10.1371/journal.ppat.1002222 (PMC3182922; doi:10.1371/journal.ppat.1002222)
Supplement: Text S1 — MS database searches of MudPIT spectra, phosphoprotein identification and statistical analysis. Summary and statistics of phosphopeptide identifications based on Sequest or Mascot database searches of MudPIT raw data, as listed in Supplementary Tables S3 and S4 (DOC) [file ppat.1002222.s019.doc]

**MS database searches of MudPIT spectra, phosphoprotein identification and supplementary statistics**

After our MudPIT runs we searched the latest non-identical LudwigNR protein database (<http://ww.ludwig.edu.au/archive/ludwigNR>) and identified a total of 1024 *Toxoplasma* proteins at a 1.8% false discovery rate (FDR) (as assessed by Sequest decoy database analysis; see Table S2). Decoy database searches using the ProLuCID and DeBunker algorithms for automatic validation of phosphopeptide identifications from tandem mass spectra [1] demonstrated significant phosphopeptide enrichment with 46% of all peptide identifications of TiO_2_-bound fractions flagged as highly confident phosphopeptide spectra (Table S3), compared to 0.2% in the TiO_2_-unbound fraction (Table S2). Detailed expert analyses of TiO_2_-bound LC-MS/MS spectra using both Sequest and Mascot-based search algorithms (Tables S2 – S4) resulted in a total of 305 non-redundant *Toxoplasma* phosphoproteins at a 0.9% false positive rate supported by 496 manually approved unique phosphopeptide identifications (Tables S3, S4). This dataset comprises 546 potential phosphorylation sites, including 424 (~78%) phospho-serine, 108 (~20%) -threonine and 11 (~2%) -tyrosine residues, respectively.

To reveal potential functions of the 305 identified phosphoproteins we also investigated GO or EC classifications, Pfam or InterPro domain alignments, as well as SignalP or TMHMM motif-predictions (for the presence of a signal peptide or trans-membrane domains) downloaded from UniPROT (<http://www.uniprot.org/>) or ToxoDB (<http://www.toxodb.org/toxo/>) databases. The largest class of *Toxoplasma* phosphoproteins identified in this study is hypothetical proteins of unknown function, and the fact that they represent 51% of the observable phosphoproteome highlights the lack of comprehensive functional annotation of the *Toxoplasma* proteome. Functional categories which are highly represented are phosphoproteins with protein-, nucleic acid-, ion-, ATP- or GTP-binding functions (101 unique proteins ~33%) and enzymes with hydrolase, transferase, kinase, oxyreductase or GTPase activities (87 unique proteins ~29%). SignalP and TMHMM predictions suggest that 59 non-identical phosphoproteins (~19%) contain a signal peptide or transmembrane domain(s). The five most highly represented Pfam or InterProt domain alignments in the MudPIT phosphoproteome are proteins with nucleotide binding-, kinase-, beta transducin/ WD40 repeat-, armadillo (ARM) repeat-, or tetratrico peptide repeat region (TPR)-like domains (Figure 2A, Table S5). Based on database searches and literature mining 42 (14%) of the 148 annotated proteins could be assigned cellular functions in signal transduction, translation initiation, intracellular protein transport, transmembrane transport or motility. This data has been integrated into ToxoDB ([www.toxodb.org](http://www.toxodb.org/)) and will be available in the September 2011 release.

**References:**

**1.** Lu B, Ruse C, Xu T, Park SK, Yates J, 3rd (2007) Automatic validation of phosphopeptide identifications from tandem mass spectra. Anal Chem 79: 1301-1310.
