# Supplementary material for: Quantitative in vivo Analyses Reveal Calcium-dependent Phosphorylation Sites and Identifies a Novel Component of the Toxoplasma Invasion Motor Complex
Source: PLoS Pathog. 2011 Sep 29;7(9):e1002222. doi: 10.1371/journal.ppat.1002222 (PMC3182922; doi:10.1371/journal.ppat.1002222)
Supplement: Text S2 — Ca2+-dependent phosphorylation of GAP45. Interpretation of SILAC-based quantitative MS data for Ca2+-dependent phosphorylation sites of GAP45, as shown in Supplementary Figure S6. (DOC) [file ppat.1002222.s020.doc]

**Ca^2+^-dependent phosphorylation of *T. gondii* GAP45**

To understand the role of calcium -dependent phosphorylation on the structure of the invasion motor we analysed phosphorylation deposition of *T. gondii* GAP45 upon calcium stimulation by ^32^P labelling, immunoprecipitation and 2D gel electrophoresis. This approach revealed that GAP45 is multiply phosphorylated at a basal state and also phosphorylated multiple times upon calcium stimulation (Figure 1E, Supplementary Figure S6A). It is evident from this analysis that there are at least 3 additional spots upon calcium stimulation suggesting three calcium-dependent phosphorylation events. Due to restricted (~52%) sequence coverage we cannot be certain of the total number of phosphorylation sites used *in vivo*.

To probe which phosphorylation sites are deposited *in vivo* upon calcium signalling, we also performed an expert analysis of our SILAC raw data to manually validate the MaxQuant results shown in Table 1 and Supplementary Table S5. This allowed us, for the first time, to accurately specify phosphorylation sites of the *Toxoplasma* GAP45 that are regulated in a calcium-dependent manner *in vivo*. No significant change in the abundance of GAP45 serine residues S153, S158, S169 was observed when the ratio of heavy to light phosphopeptide intensities was normalized for GAP45 (average TgGAP45 H/L ratio = 1.8 (Table 1, Supplementary Table S5). By contrast, our quantitative proteomics analyses indentified up to three modifications of the same peptide sequence (VAEHSSAAVTDR), potentially phosphorylated at serine residues S184, 185 and threonine residue T189, that appear to be regulated by Ca^2+^-dependent phosphorylation pathways. The MaxQuant algorithm calculated a normalized increase in the H/L ratio of 3.9- and 3.7-fold for modifications on serine or threonine residues, respectively (Table 1, Supplementary Table S5). However, the Mascot search engine was unable to distinguish between the phosphorylation of S184/5 (ΔMascot = 0.9) and MaxQuant-based quantification of this peptide appeared to be coupled with significant experimental variation (Tables 1, S5). Manual inspection of the extracted ion chromatograms (XICs) of light and heavy-labelled SILAC peptides at m/z(L)=661.79 and m/z(H)=664.79 showed three peaks eluting within half a minute (Figure S6B). This data indicates the presence of three distinct phosphopeptide ions at RT=38.43, RT=38.72 and RT=38.85. The MS/MS spectrum taken at RT=38.39 unambiguously identified the corresponding ion as phosphopeptide VAEHSSAAVT(ph)D (Figure S6D). Based on the integrated raw intensity of the SILAC pair across peak 1 (RT:38.38 - 38.48; Figure S6B) we estimated a H/L ratio of this phosphopeptide of ~6.3 (Figure S6C), which in accordance with the value estimated by MaxQuant to a normalized H/L ratio of 3.7 (Tables 1, S5). The same was true for the second and third peak which yielded a normalized H/L ratio of ~ 3.9 (Table 1, Figure S6C). However, if the two peaks were integrated separately we determined a normalized H/L ratio of ~7.0 for peak 2 (RT: 38.72) and ~2.8 for peak 3 (RT: 38.85), respectively (not shown). Manual interpretation of the MS/MS fragmentation spectrum acquired at RT=38.87, revealed fragment ions with phospho-modification at S184 (b5 +80) as well as neutral loss of H_3_PO_4_ at S185 (y7-98^2+^) (Figure S6D, arrows). This result is consistent with a mixed population of parent ions being phosphorylated on S184 or S185 (ie.VAEHS(ph)SAAVTD and VAEHSS(ph)AAVTD). Due to dynamic exclusion of the doubly charged ion we failed to obtain discrete MS/MS localization data for peak 2 in our SILAC experiments, but we also observed multiple MS/MS fragmentation spectra that provided strong evidence for phosphorylation of S184 from our MudPIT analysis (Figure S2E). Due to technical limitations we were unable to discriminate and therefore accurately quantify the presumably co-eluting VAEHS(ph)SAAVTD and VAEHSS(ph)AAVTD phosphopeptides. Selective ion monitoring using a triple quadrupole linear ion trap mass spectrometer may facilitate conclusive quantitative analyses in future. Together, the present quantitative measurements of GAP45 phosphorylation confirmed at least four Ca^2+^-independent residues (S153, Y158, S163, S169) and identified up to three serine/threonine residues (S184, 185 and T189) that are major targets of Ca^2+^-dependent phosphorylation pathways.
