# Supplementary material for: Quantitative in vivo Analyses Reveal Calcium-dependent Phosphorylation Sites and Identifies a Novel Component of the Toxoplasma Invasion Motor Complex
Source: PLoS Pathog. 2011 Sep 29;7(9):e1002222. doi: 10.1371/journal.ppat.1002222 (PMC3182922; doi:10.1371/journal.ppat.1002222)
Supplement: Text S3 — Ca2+-dependent phosphorylation of MLC1. Interpretation of SILAC-based quantitative MS data for Ca2+-dependent phosphorylation sites of MLC1, as shown in Supplementary Figure S7. (DOC) [file ppat.1002222.s021.doc]

**Ca^2+^-dependent phosphorylation of TgMLC1**

*In vitro* evidence in *P. falciparum* indicates that both PfGAP45 and PfMTIP are substrates for PfCDPK1 [1], but there is currently no information on Ca^2+^-dependent phosphorylation of *T. gondii* MLC1. Computational analyses using the MaxQuant algorithm demonstrated no significant difference in the H/L ratio of S55-modified phosphopeptide compared to a 1.6- to 1.7-fold increase in the relative abundance of phosphopeptides modified on phosphorylation sites T98 or S132, when normalized for an estimated 2.8-fold increase estimated for all MLC1 peptides (see Table 1, Supplementary Table S5). Figure S7A shows the extracted XICs for the light or heavy-labelled SILAC ion pairs of MLC1 phosphopeptides VGEYDGACES(ph)PSCR, VST(ph)GDAMILAR, and SGDNLDYAS(ph)FQK. These phosphopeptides were generally well resolved as doubly charged ions at RT=41.41, RT=49.57, and RT=48.80, with relative intensities of the respective SILAC pairs ranging between H/L~2.7 for S53 to H/L~4.5 for S132 (Figure 7B). These values are in accordance with the normalized computational estimates reported in Tables 2 and S5. The manually annotated MS/MS spectra for associated parent ions also identified modified b and y ions that unambiguously localized the different phosphorylation sites (Figure 7C). Together, our SILAC data indicate little or no change in the phosphorylation of *T. gondii* MLC1 residue S53 and confirm Ca^2+^-dependent regulation of phosphor-modifications at residues T98 and S132 that may potentially be involved in the control of invasion motor activity in *Toxoplasma*.

**References:**

**1.** Green JL, Rees-Channer RR, Howell SA, Martin SR, Knuepfer E, et al. (2008) The motor complex of *Plasmodium falciparum*: phosphorylation by a calcium-dependent protein kinase. J Biol Chem 283: 30980-30989.
